# Geography of current and future global mammal extinction risk

**DOI:** 10.1371/journal.pone.0186934

**Published:** 2017-11-16

**Authors:** Ana D. Davidson, Kevin T. Shoemaker, Ben Weinstein, Gabriel C. Costa, Thomas M. Brooks, Gerardo Ceballos, Volker C. Radeloff, Carlo Rondinini, Catherine H. Graham

**Affiliations:** 1 Department of Ecology and Evolution, Stony Brook University, Stony Brook, New York, United States of America; 2 NatureServe, Arlington, Virginia, United States of America; 3 Department of Natural Resources & Environmental Science, University of Nevada, Reno, Nevada, United States of America; 4 Department of Biology, Auburn University at Montgomery, Montgomery, Alabama, United States of America; 5 International Union for Conservation of Nature, Gland, Switzerland; 6 World Agroforestry Center, University of the Philippines Los Baños, Laguna, Philippines; 7 Institute for Marine and Antarctic Studies, University of Tasmania, Hobart, Tasmania, Australia; 8 Instituto de Ecologia, Universidad Nacional Autonoma de Mexico, México D.F., México; 9 Department of Forest and Wildlife Ecology, University of Wisconsin-Madison, Wisconsin, United States of America; 10 Global Mammal Assessment program, Department of Biology and Biotechnologies, Sapienza University of Rome, Roma, Italy; 11 Unit of Biodiversity and Conservation, Swiss Federal Research Institute, Birmensdorf, Switzerland Unit of Biodiversity and Conservation, Swiss Federal Research Institute (WSL), Birmensdorf, Switzerland; University of Massachusetts Amherst, UNITED STATES

## Abstract

Identifying which species are at greatest risk, what makes them vulnerable, and where they are distributed are central goals for conservation science. While knowledge of which factors influence extinction risk is increasingly available for some taxonomic groups, a deeper understanding of extinction correlates and the geography of risk remains lacking. Here, we develop a predictive random forest model using both geospatial and mammalian species’ trait data to uncover the statistical and geographic distributions of extinction correlates. We also explore how this geography of risk may change under a rapidly warming climate. We found distinctive macroecological relationships between species-level risk and extinction correlates, including the intrinsic biological traits of geographic range size, body size and taxonomy, and extrinsic geographic settings such as seasonality, habitat type, land use and human population density. Each extinction correlate exhibited ranges of values that were especially associated with risk, and the importance of different risk factors was not geographically uniform across the globe. We also found that about 10% of mammals not currently recognized as at-risk have biological traits and occur in environments that predispose them towards extinction. Southeast Asia had the most actually and potentially threatened species, underscoring the urgent need for conservation in this region. Additionally, nearly 40% of currently threatened species were predicted to experience rapid climate change at 0.5 km/year or more. Biological and environmental correlates of mammalian extinction risk exhibit distinct statistical and geographic distributions. These results provide insight into species-level patterns and processes underlying geographic variation in extinction risk. They also offer guidance for future conservation research focused on specific geographic regions, or evaluating the degree to which species-level patterns mirror spatial variation in the pressures faced by populations within the ranges of individual species. The added impacts from climate change may increase the susceptibility of at-risk species to extinction and expand the regions where mammals are most vulnerable globally.

## Introduction

Human impacts are causing widespread biodiversity loss, with rates of extinction that are about 1,000 times greater than background levels [1–3]. One-fifth of all vertebrates are threatened with extinction [4]. With human population expected to grow from 7.6 billion to more than 9 billion over the next few decades, and consumption rising even faster, humanity’s impact on the planet’s biodiversity is projected to increase substantially [5]. Knowledge of which species are at greatest risk, why, and where they are most vulnerable is consequently a central goal for conservation science.

Yet, large gaps in our knowledge of species threat exist, even for well-studied taxa [6]. For example, assessment of risk under the IUCN Red List of Threatened Species has only been completed for 66% of all vertebrates, of which 15% are assessed as Data Deficient (DD), lacking sufficient information to determine their conservation status [7,8]. Further, while factors influencing extinction risk have been identified [9–11], we lack systematic investigation of the geographic patterns of important risk factors in some geographic regions but not others [12,13]. Uncovering the statistical and geographic distributions of trait, environmental, and threat variables that predict species-level risk is important for understanding the underlying nature and global geographies of extinction correlates. Take the well-known extinction driver, small geographic range size, as an example: What are the geographic range sizes that emerge as being most associated with risk, and where does this factor have the most influence across the globe? New ecoinformatic and spatial databases make it now possible to identify these macroecological relationships and go beyond our current understanding of extinction risk. Finally, we do not know which species will be at greatest risk in the future, and where they will be most vulnerable. While human land use is the major driver of species extinctions today, climate change is a growing and compounding threat, with climate zones projected to shift across ca. 20% of the Earth’s land surface by the end of this century [14,15].

Predictive models of threat can help fill in information gaps. For example, they can be used to identify intrinsic traits that make species especially at risk, such as geographic range, body size, and speed of life history [9,10,16]. Risk also can be predicted on the basis of extrinsic factors relating to susceptibility to different threat types, such as human population density, habitat loss, over-exploitation, and climate change [17,18]. Species with certain biological traits can be more susceptible to particular threat types than others and these threats vary temporally and spatially [19–21]. Using a random forest predictive modelling approach, as we do in this study, allows us to identify and account for such important interactions in parameter space [for example, species with small geographic ranges may be more likely to be at risk from a particular extinction driver than species with large geographic ranges [9,10]]. Predictive models are especially valuable to inform the status of Data Deficient species, as well as to understand which species are currently not assessed as threatened but have intrinsic traits and geographic distributions which render them likely to become threatened in the future [hereafter, “latent risk”, see [22]]. As human impacts expand and increase, these species facing latent risk are among those most likely to become threatened [22].

Climate change poses a particular challenge. This is because the time horizon of established species conservation assessments, like the IUCN Red List, that are often used to train predictive extinction risk models is often shorter than that over which the impacts of climate change on species risk are expected [23]. However, many of the traits that make species susceptible to extinction in general also make them vulnerable to climate change impacts, and so the Red List can be informative in assessing risk under climate change [24–26].

Here, we address these gaps in our understanding of global mammal extinction risk and go beyond previous studies focused solely on identifying risk correlates, by uncovering the statistical and geographic distributions of extinction correlates and exploring how the landscape of risk may change under a warming climate. We used a random forest modeling framework and geospatial and trait data on all mammals to identify: (i) the drivers of *why* are species at risk, identifying key intrinsic (i.e., biological) and extrinsic (i.e., environmental) correlates, including the range of values for each variable that are associated with risk; (ii) *where* species that are threatened by a given extinction driver are concentrated across the globe; (iii) *which* species are potentially (predicted to be) at risk, in addition to those actually assessed as threatened on the IUCN Red List, and where they occur; and (iv) *how* species might be impacted by a rapidly changing climate.

## Materials and methods

We compiled a species-level database for 4,864 mammals, excluding cetaceans [16,27]. We collected data on intrinsic biological traits: adult body mass, geographic range size, mass-specific production (i.e., speed of life history) [28], social group size, trophic group, activity cycle, home range size, population density, habitat mode, landmass type, taxonomic order [16,27]. Note that geographic range and landmass type are the result of interactions between both intrinsic species traits and extrinsic environmental variables; we treat them here as intrinsic traits for simplicity. We gathered additional data on extrinsic environmental variables: cumulative annual productivity, minimum annual productivity (i.e., harshness of environment), variation in annual productivity (i.e., seasonality), land cover type (including human land use) [29] (S1 Fig), median latitudinal and longitudinal position across a species range, human population density [30], and night-time lights [31], within each species’ geographic range. The extrinsic productivity variables were based on the Dynamic Habitat Index [DHI; [32]], newly calculated here for the globe. DHI consists of three indices extracted from an annual series of monthly Moderate Resolution Imaging Spectroradiometer (MODIS) Fraction of Photosynthetically Active Radiation (fPAR) data for the period of 1981 to 2011. The three indices summarize the minimum, accumulated, and annual variation in satellite measured primary production [32]. So, these indices represent environmental harshness, total annual primary production, and seasonality, respectively, which yield a more complete picture of environmental conditions than typical productivity measures, such as Net Primary Productivity (NPP) [32]. Our species distribution maps were in the form of pixels of suitable habitat inside known geographic ranges at 300 m resolution [33]. We assessed high habitat suitability on the basis of land cover, elevation, and presence of water, reclassified according to the habitat relationships identified in the IUCN Red List [33]. For each extrinsic variable used in our model, we extracted the average, maximum, and minimum values across each species’ range at 0.25 degree, using the Raster package in R [34]. Because our preliminary random forest models found that mean values of extrinsic variables were the best predictors of risk, rather than maximum or minimum values, we used only mean values in this study, a similar approach to that used by Davidson et al. [11]. For categorical variables, we assigned each species the modal value within its geographic range.

Some of the intrinsic traits, such as geographic range size, that we used to build our model are similar to those used as input data into IUCN Red List species’ assessments. This would introduce circular reasoning if our primary objective was to identify and rank the true underlying drivers of extinction risk. However, our goal here was to train our model using the IUCN Red List so that we could uncover underlying critical ranges and geographic patterns of traits associated with risk, and to identify species that are not currently known to be at risk but might be vulnerable because they share similar traits and environments with those on the IUCN Red List. Because extrinsic environmental variables are not used to inform Red List assessments, we also evaluate their relative importance as correlates of IUCN Red List category, in addition to identifying the statistical and geographic patterns associated with risk.

We used a dichotomous response variable to represent risk: species assessed as Vulnerable (V), Endangered (E), Critically Endangered (CR), Extinct in the Wild (EW), or Extinct (EX) on the IUCN Red List were considered “at risk”, and species assessed as Near Threatened (NT) and Least Concern (LC), were considered “not at risk” [35]. This dichotomous classification provided a more powerful and accurate analysis of extinction risk than when all IUCN Red List categories were considered separately, because relatively few species belong to each of the different threatened species categories while comparably large numbers of species are non-threatened.

We quantified relationships between predictor variables and extinction risk using a random forest model of 500 conditional inference trees [36,37]. Random forest models are a machine learning, recursive partitioning technique that combines the predictions of multiple independent trees into a robust composite model, with high predictive accuracy [36,38,39] and the ability to implicitly deal with non-linear, context-dependent interactions among multiple, correlated predictor variables [36,40]. Random forest models can also provide viable alternatives to phylogenetic contrasts [37,41,42]. Following similar studies [11,43], we included taxonomic order in our models to account for phylogeny as a determinant of extinction risk. Our modelling framework was similar to Davidson et al. 2009 and 2012, but here we used conditional inference forests, in the "party" package in R, a distribution-free random forest model that can improve predictive performance, corrects a known bias in conventional recursive partitioning methods [44], and has the ability to handle missing data (so imputation is not required).

We used the random forest model output to predict threat status for each species, including for DD species (S1 Table), and to estimate the relative importance of each predictor variable (S2 Fig). Raw model results were in the form of probabilities (i.e., estimated probability of each species being assessed as "threatened" under IUCN Red List). To predict the binary threat status of each species (e.g., "Data Deficient" species), we classified species exceeding the maximum kappa threshold [threshold associated with the maximum Cohen's Kappa statistic [45]] as "at risk". We assessed model performance using standard 10-fold cross-validation, in which each of 10 approximately equal data partitions ("folds") were held out in turn from the analysis while the remainder was used for model training. Model performance was assessed using area under the curve (AUC) from the Receiver Operating Characteristic (ROC), and (for the threshold results) total classification accuracy, sensitivity, specificity, and Cohen's Kappa (S2 Table; S4 Fig).

**Fig 1:**
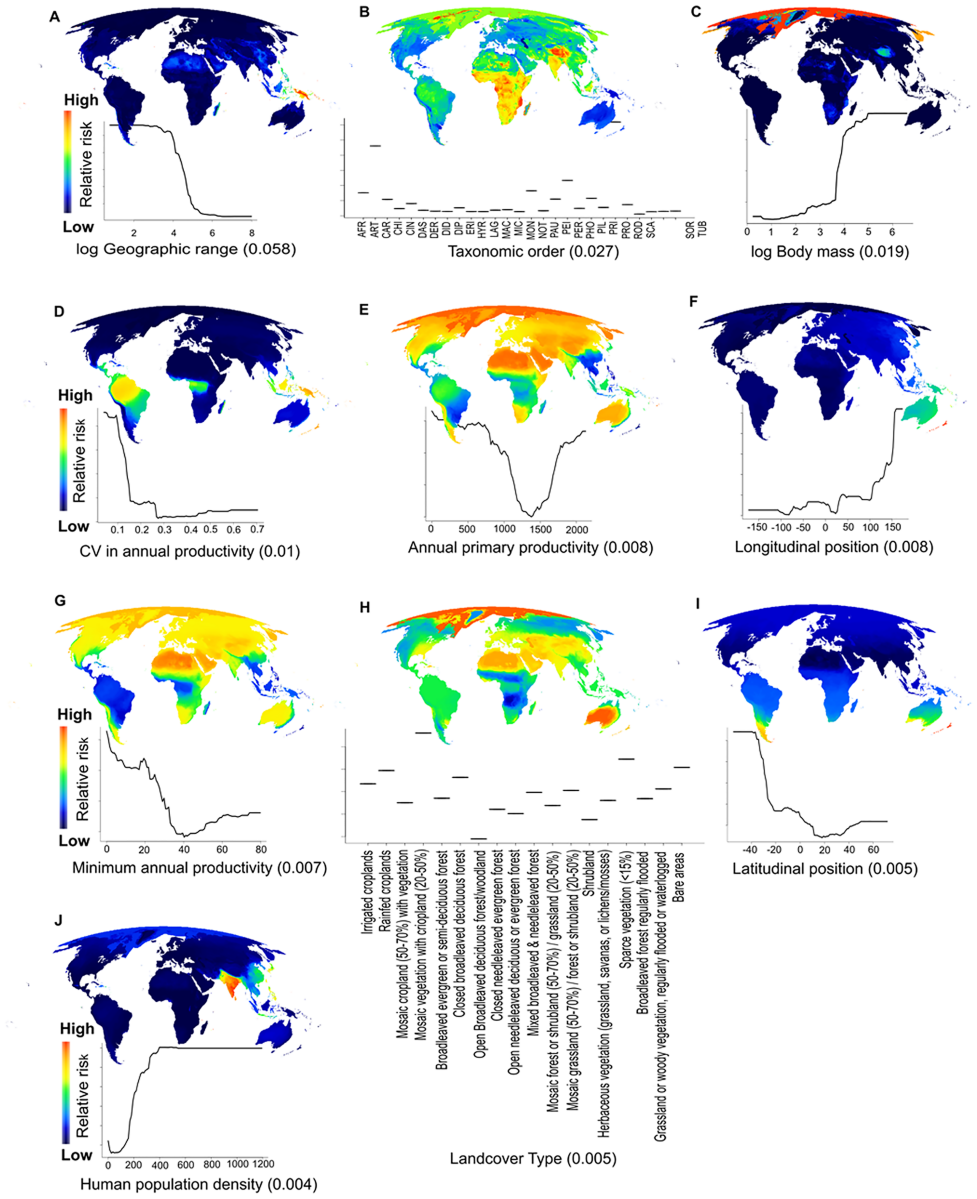
Expected contributions of each extinction correlated with global mammal extinction risk across geographic space, and the range of values most associated with risk for each predictor. Species at high risk of extinction due to a particular variable are more concentrated in red areas than those in blue. Importance values are indicated in parentheses under each univariate plot, and predictors are displayed in their relative order of importance. (*A*) Log geographic range size (km^2^). (*B*) Taxonomic order; orders are abbreviated with the first three letters. (*C*) Log body mass (grams). (*D*) Coefficient of variation in annual primary productivity (i.e., seasonality). (*E*) Total annual primary productivity. (*F*) Longitudinal position. (*G*) Minimum annual primary productivity (i.e., environmental harshness). (*H*) Landcover type. (*I*) Latitudinal position. (*J*) Human population density (number/km^2^).

We generated partial dependence plots (shown in Fig 1) from the random forest model, to display the relationship of each predictor variable with risk when all other variables were held at their means. The plots show the statistical relationships between predictors and risk that emerge from the random forest model. By doing so, they highlight the functional form of the relationship between each variable and extinction risk, including points where extinction risk rises sharply.

To gain insight into where species tended to be at greatest risk from each of the most important intrinsic and extrinsic predictor variables, developed maps of the univariate contributions of each extinction driver to species-level risk (Fig 1). To accomplish this, we used the random forest model to predict extinction risk for each species based only on the observed values of the focal variable, holding all other variables at their global mean values. For each predictor variable, we then computed the mean risk of extinction across all species known to occur in each raster pixel, showing the geographic locations at which species were predicted to be at highest risk of extinction due to each respective risk factor.

**Fig 2:**
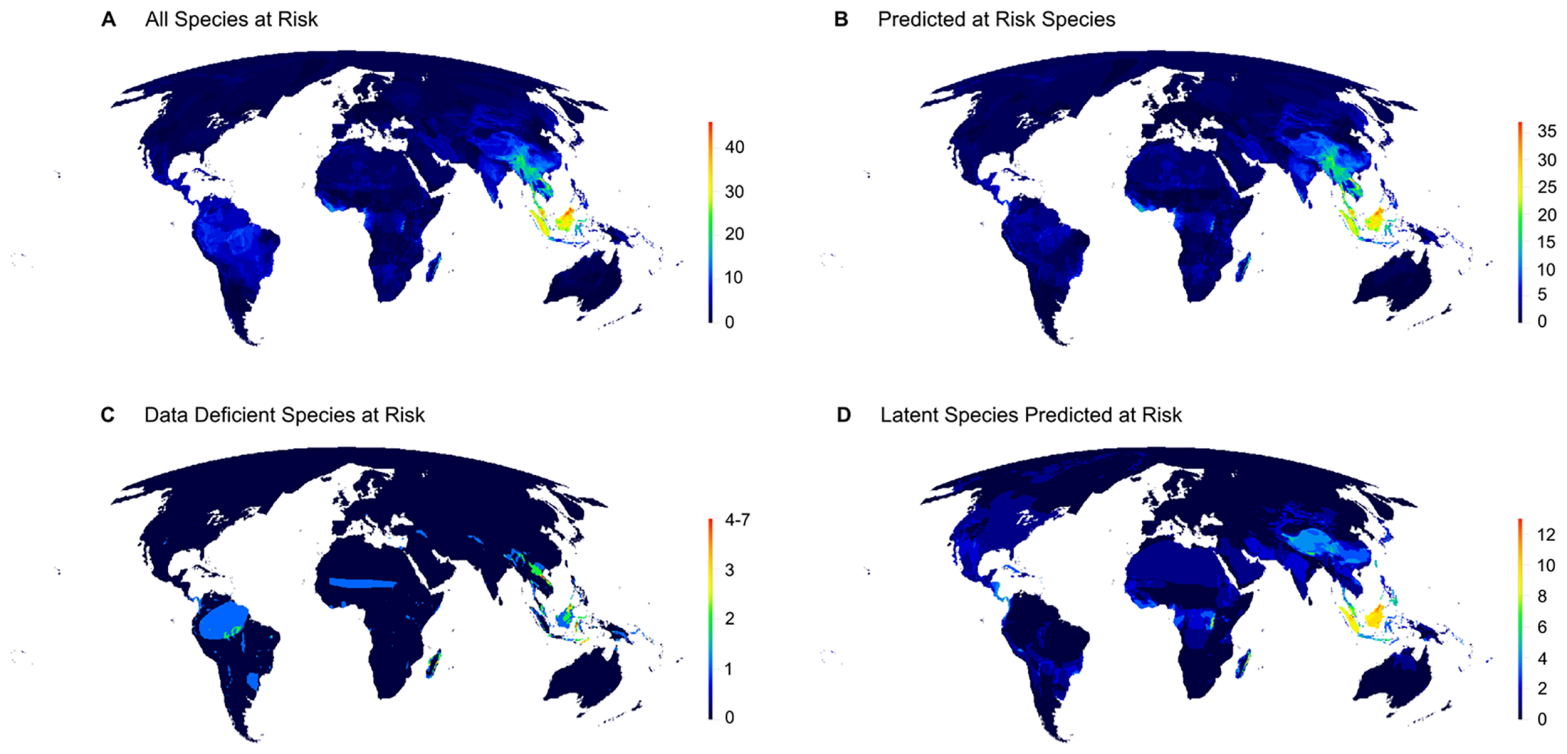
Geographic distribution of global mammal extinction risk. (*A*) All species actually or potentially at risk (species actually assessed as threatened on the IUCN Red List, plus those predicted to be at risk). (*B*) All species predicted to be threatened. (*C*) Data Deficient species predicted to be threatened. (*D*) All species predicted to be at latent risk (species not currently assessed as threatened on the IUCN Red List, but predicted to be at risk of extinction by our model). Panel C is a subset of panel D, which is a subset of B, which in turn is a subset of A.

**Fig 3:**
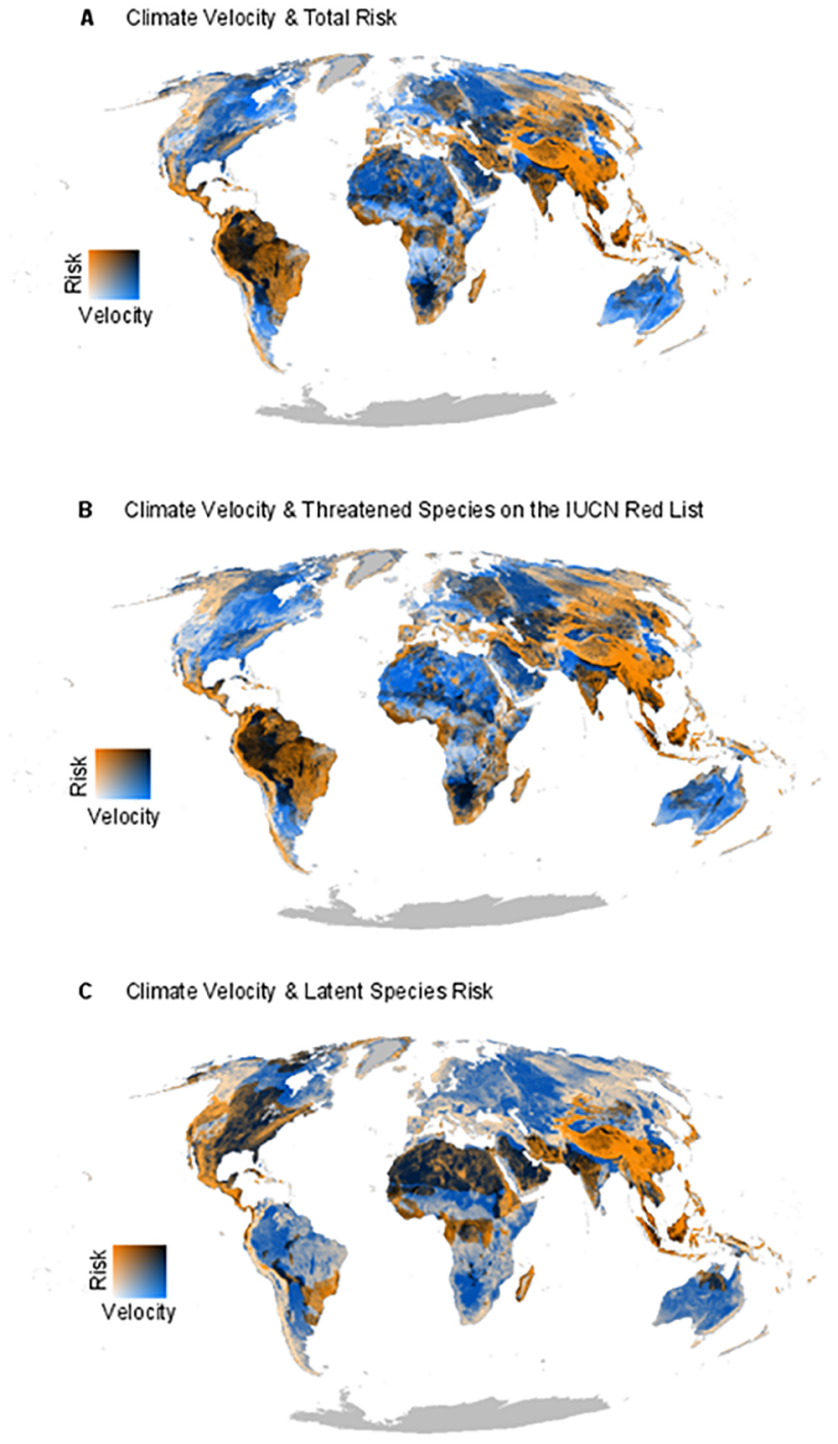
Velocity of climate change and global mammal extinction risk. Spatial overlay of the velocity of climate change with: (*A*) all actually and potentially at risk species (species assessed as at risk on the IUCN Red List and species predicted to be by our model); (*B*) only actually at risk species (species currently assessed as threatened on the IUCN Red List); and (*C*) latent species risk (species not currently assessed as threatened on the IUCN Red List but are predicted to be at risk of extinction according to our model).

To visualize the spatial distribution of mammal species at risk globally, we overlapped geographic ranges of mammal species at risk and then counted how many of them were found in each quarter-degree grid cell (Fig 2). We created maps that show risk across four different perspectives: 1) *All species actually or potentially at risk* [those that are already on the IUCN Red List as threatened, and those predicted to be at risk according to our model (including DD species) but not assessed as threatened on the Red List (“latent risk”)] (Fig 2A); 2) *Predicted to be at risk* [all species at risk according to our model (including DD species)] (Fig 2B); 3) *Data Deficient species predicted to be at risk* (those assessed as DD on the Red List) (Fig 2C); 4) *Latent species predicted at risk* [species predicted to be at risk according to our model (including DD species) that are not assessed as threatened on the Red List (“latent risk”)] (Fig 2D). We refer throughout the manuscript to species “actually at risk” as are those that are assessed as (VU, CR, EN, EW, EX) on the Red List, and species that are “potentially at risk” as those that are not identified as at risk on the Red List but are predicted to be according to our model (this includes DD species predicted to be at risk).

Finally, we evaluated the spatial overlap of present-day extinction risk and predicted latent risk with global climate change velocity, a measure of the speed at which climate is changing based on the instantaneous horizontal velocity of temperature change between 2050–2100 [46](Fig 3). We used climate change velocity rather than a set of climate change model scenarios, because the velocity of climate change is more closely linked to a species’ ability to adapt to changing climate or migrate to suitable climates [46]. We overlaid the distributions of species that are biologically and geographically most vulnerable to extinction, with those areas that are predicted to undergo the most rapid pace of climate change [10,47].

## Results

### Why species are actually or potentially threatened

In our model, biological traits were the most important predictors of risk (Fig 1 and S2 Fig), especially geographic range size, followed by taxonomic order, body mass, and landmass type (i.e., island and/or mainland). Although extrinsic variables were less important in the model, they were still important drivers of extinction risk. The most important environmental variables, respectively, were: annual change in plant primary productivity (i.e., seasonality), total annual productivity and minimum productivity (i.e., environmental harshness), geographic location (latitude and longitude of geographic range centroids), land cover/land use type, and human population density (Fig 1). We also found that each extinction correlate exhibited distinct statistical distributions of values that were strongly associated with risk, including the points where extinction risk rises sharply (Fig 1). These points may represent critical thresholds of risk, values beyond which the risk of extinction is extremely high.

### Where species at risk from different extinction drivers are concentrated across the globe

The occurrence of species at risk due to different extinction drivers exhibited wide geographic variation across the globe. Globally, species at risk due to small geographic range size were most commonly found in Southeast Asia, while taxonomic orders at greatest risk (i.e., primates, artiodactyls, and perissodactyls) were most concentrated in Africa, Madagascar, and parts of Asia and the Arctic. Species most at risk due to large body size were especially concentrated in the Arctic. At risk species for which low seasonality was an important predictor were concentrated in the world’s tropical regions; whereas, those with low annual primary productivity and harsh climate as important predictors occurred mostly in high latitude and inter-continental regions. Species whose risk correlated with latitude and longitude in our models were concentrated in Australia, New Zealand, and the southern tip of South America. Landcover, in particular mosaic vegetation with crop land, sparse vegetation, bare areas, rainfed croplands and closed broadleaved deciduous forest, is an important predictor for species that occur Australia and to a lesser extent much of southern Asia, the Sahara Desert and eastern North America. Finally, human population density played the greatest role as a predictor of risk for species concentrated in Southeast Asia.

### Which species are actually or potentially threatened and where they occur

Our random forest model predicted species extinction risk on the IUCN Red List, with 83% accuracy (Cohen's kappa = 0.5; see S2 Table and S3 and S4 Figs and for all goodness-of-fit metrics). Overall, our model characterized 457 mammal species (10% of mammal species considered) as at risk that are currently not assessed as threatened, based on intrinsic and extrinsic factors. Of these, 253 species (5% of mammal species included) are currently assessed as NT or LC. These species can be considered to face latent risk. Using our model to predict the conservation status of DD species, we found that 204 (36% of DD species) have intrinsic traits and extrinsic factors associated with high extinction risk (S1 Table). We found Southeast Asia had, by far, the greatest number of actually and potentially threatened species (Fig 2).

### How species might be impacted by future climate change

Many species actually or potentially threatened are likely also to experience relatively rapid climate change (1,379; S1 Table). Nearly 40% (537) are predicted to experience velocities of change at 0.5 km/year or more, and 17% (226) could experience changes at 1 km/year or more, averaged across their ranges. The map of climate change velocity [46], overlaid with our maps of both current and latent extinction risk, showed a suite of geographic areas that are rapidly warming and harbor many actually or potentially threatened species (Fig 3). This included much of Southeast Asia, which exhibited even greater risk when also considering the rate of changing climate in this region. But, our results also showed that high-risk regions might extend well beyond Southeast Asia in the future (Fig 3A and 3B).

## Discussion

### Why species are at-risk and where different factors are threatening

Our work differs from previous studies by uncovering the distinct statistical and geographic distributions of both intrinsic traits and extrinsic environmental variables associated with risk (Fig 1). While biological traits are well-known *intrinsic* correlates of risk [e.g., [9,11,16,20]], their importance as predictors is not the focus of our work. Instead, we illuminate the statistical range of values and geography of biological traits, whereby species are most vulnerable to extinction (Fig 1A–1C). We also identify and disentangle key *extrinsic* correlates of risk; some of the most important ones in our model have not been evaluated as extinction correlates before, such as those that represent seasonality and environmental harshness [10,20,43,48], offering new information on extrinsic drivers of risk. The macroecological patterns we identify provide new insights into the quantitative relationships that exist between risk and extinction correlates.

#### Intrinsic traits

Geographic range and body size are recognized as among the most important predictors of mammalian risk [9,10]. While biological traits and geography underlie species’ geographic range sizes, today, range sizes are also the product of human pressures [49]. We found that species with geographic range sizes greater than about 7,700,000 km^2^ were predictably at low risk, such as the wide-ranging deer mouse (*Peromyscus maniculatus*) in North America. Whereas, risk rapidly increased for species with range sizes smaller than that, especially below around 1,000,000 km^2^ (Fig 1A). The hairy-nosed otter (*Lutra sumatrana*) in Southeast Asia has a geographic range size just below 1,000,000 km^2^, for example, and is currently listed as Endagered under the IUCN Red List and was predicted by our model to be at risk. Many of these species occur on islands, especially in Oceania. We also found that species with body sizes larger than about 8.6 kg exhibited higher risk, so these would be species that are larger than about the size of a fox. This body size (> 8.6 kg) where risk increases is a few kg (~25%) higher than, but still broadly consistent with, previous studies [10,16]. The Arctic harbored a proportionally high number of large body sized mammals associated with risk (Fig 1C), which may reflect the geographical association of large body sized mammals with colder climates [50], and the recent vulnerability of many of these species to climate change [51,52]. This finding differs from Fritz et al. [13] who found large body size was a key correlate of species risk in tropical regions, apparently reflecting historical declines of large bodied species.

While taxonomy can be another correlate of risk [53,54], we found that its contribution was not uniform across the globe. Primates, artiodactyls, and to a lesser extent perissodactyls, were the orders predicted to be most at risk, and these orders were most concentrated in Africa and Madagascar and parts of Asia and the Arctic (Fig 1B). Orders associated with high extinction risk were those that had large body sized species with slow life histories; many of these species are highly threatened by over-hunting, illegal wildlife trade, and habitat loss [10,55–57]. Taxonomic order as a predictor variable may also serve as a proxy for a suite of other factors that we did not include in our model.

#### Extrinsic variables

Our model highlights the importance of geographic setting on extinction risk. Species that live in environments with low seasonality (i.e., where the coefficient of variation in annual productivity was low) and that have high primary productivity are more likely to be threatened (Fig 1D, 1E and 1G). These areas mostly represent tropical, biodiverse regions that harbor numerous species with small geographic range sizes and narrow niche breadths, and are experiencing high levels of human activity, especially deforestation and over-hunting [57]. This may reflect the common pattern that areas that are ideal for human settlement are often also high in biodiversity [58]. However, species that live under naturally harsh environmental conditions (i.e., where minimum annual productivity and overall primary productivity are low) were also at high risk. Harsh climate was a key predictor of risk for species that occur throughout much of the planet’s semi-arid, arid, and cold environments (Fig 1E and 1G). Animals living in these physiologically demanding environments may be less able to withstand additional stress caused by human activities. Survival and offspring production can be highly variable in these environments [59], and human impacts can have compounding effects, such as overexploitation, habitat loss, and more frequent environmental extremes due to climate change [60].

Longitude and latitude also were important predictors of risk in our model [see also [10]], with species occurring at longitudes beyond 140 degrees east and less than 20 degrees south being especially at risk (Fig 1F and 1I). Species whose risk correlated with latitude and longitude in our models were concentrated in Australia, New Zealand, and the southern tip of South America. Latitude and longitude undoubtedly functioned as proxies for other extinction risk drivers that were not included in our model. One possible factor is introduced predators, which threaten native bats in New Zealand and have devastated populations of mammals in Australia [61]. In fact, although Australia has only 6% of the world’s mammal species, one-third of mammal extinctions since 1500 have occurred in Australia, largely due to introduced predators, as well as habitat degradation and introduced herbivores [61,62]. Most Australian mammal declines have occurred in the drier, southern portion of Australia and increasingly northern species are showing rapid declines [61]. Similar proxies may explain at-risk species in the southern tip of South America, which harbors the southern-most mammals of the world. For example, in the region’s temperate forests, the southern pudú (*Pudu puda*, VU) is threatened by introduced predators and competition with domestic livestock, and the kodkod (*Leopardus guigna*, VU) is threatened by deforestation and predator extermination [63].

Geographic distribution of risk also varied among different habitat and land cover types [Fig 1H and S1 Fig; see also [57]]. The land cover types most associated with risk were natural and semi-natural terrestrial areas mixed with cropland, which are largely human-dominated landscapes. Sparsely vegetated or bare areas also were associated with high risk. Even though many of these desert and high mountain areas are remote and relatively wild, many of the species in these environments face high risk [59]. This result is consistent with our other environmental measures of risk indicating harsh environment as an important predictor of risk. Rainfed croplands and closed deciduous forests were the land cover types also associated with risk. About 80% of the world’s croplands are rainfed agriculture, which is largely industrial-scale agriculture in developed countries and the mainstay of subsistence farming in developing countries [64,65]. Species with large proportions of rainfed cropland within their range likely have experienced widespread habitat loss. Many of the closed deciduous forests occur in the eastern United States and Europe, where they have a long history of being extensively harvested, managed, and populated [66].

High human population density is a well-known predictor of risk, because it is often associated with overhunting, human-wildlife conflicts, and habitat loss [22,56,67–69]. We found that risk rapidly increased above 200 people per km^2^, and reached a critical threshold at 400 people per km^2^, beyond which all species were predicted to be at risk (Fig 1J). Species threatened by high human population density are concentrated in Southeast Asia, especially India (Fig 1J), where human population densities are high over large areas [70]. Much of Southeast Asia is undergoing rapid population and economic growth, especially India and China, and mammals throughout the region are experiencing widespread habitat loss from competition with humans for available habitat, deforestation, and cultivation, and devastating losses from overhunting for local consumption and wildlife trade [67,68]. India, for example, harbors the largest remaining populations of tigers (*Panthera tigris*, EN), but even in protected areas tigers are threatened by growing pressures from commercial interests (mining, roads), local community resource needs, declining prey availability, and poaching to sell their body parts to Asian markets [71]. Southeast Asia also is the global center for wildlife trade; in just over a 10-year period (1998–2007) at least 0.4 million mammals were officially exported from this region, and this number does not include unregulated species or those exported illegally [72].

### Which species are actually or potentially threatened and where they occur

We show that ~10% of mammals not currently recognized as threatened (457 species) have biological traits and occur in environments that predispose them towards extinction. Most latent risk and potentially threatened DD species were small mammals and species with small geographic ranges; others have similarly found that many DD species at risk have small geographic range sizes [16,43]. While our model predicted 36% of DD species to be at risk, this proportion of DD species predicted to be at risk has varied among studies, perhaps due to underlying differences in datasets and analytical approaches [16,43]. In our analysis, latent risk and potentially threatened DD species included bats, rodents, and artiodactyls in Southeast Asia, such as the Obi Island rat (*Melomys obiensis*, LC) and the Taiwan serow (*Capricornis swinhoei*, LC), which we identified to be at latent risk, and the Gaskell's false serotine bat (*Hesperoptenus gaskelli*, DD) that is only known from one location on Sulawesi. Latent risk and potentially threatened Data Deficient species also included a number of shrews and primates. These included the jackass shrew (*Crocidura arispa*, LC) that is endemic to Turkey and known from only two localities, and the recently described Lariang tarsier (*Tarsius lariang*, DD), again, from Sulawesi.

Many species actually or potentially threatened occur in areas projected to experience rapid climate warming. Indeed, many of them are already considered Endangered or Critically Endangered, suggesting that climate change will have compounding impacts. The Pacific sheath-tailed bat (*Emballonura semicaudata*, EN) is projected to experience the highest velocity of climate change on average within its restricted, and heavily impacted, geographic range of 5,000 km^2^. More broadly, the species that occur in regions with the highest projected climate velocities are those with small geographic ranges, including a number of island endemic bats in Indonesia, the sea otter (*Enhydra lutris*, EN) along the North Pacific coast, the narrow-faced kangaroo rat (*Dipodomys venustus*, LC) along the coastal mountains of west-central California, and a suite of primate species in West Africa (S1 Table).

Our results underscore the urgent need for conservation efforts in Southeast Asia (Fig 2) [see also [57,73,74]]. The IUCN Red List identifies this region as having the greatest number of mammals threatened with extinction [57]. Our model also showed that many of the species predicted to be at risk in this region are not currently on the Red List (i.e., latent risk and DD species). Overall, Southeast Asia has so many actually and potentially threatened species (Fig 1A) because it harbors many island and endemic species with small geographic ranges, has high human population densities, rampant illegal wildlife trade, and rapid deforestation -especially for palm oil plantations, that continues largely unabated [57,68]. Our model showed that many species in Southeast Asia have intrinsic traits that make them vulnerable, and these traits combined with external factors create an area of high conservation risk. Compounding this, the region harbors a large number of evolutionary distinct species that are currently threatened (i.e., “EDGE species”) [73]. However, many species in Southeast Asia have a high likelihood of recovering if pressures were eased [75], reinforcing its importance for conservation action. Other regions of risk included Central America, Amazonia, and parts of Africa. Latent risk revealed some regions where relatively few species are currently threatened [57], including in North America, the Sahara, the Arabian Peninsula, and the Congo (Fig 2).

### How species might be impacted by future climate change

Climate change will increasingly affect species in the future, due to changing and potentially novel climates [14,15]. And, the rate of climate change is projected to increase with rising global temperature, so the pace at which species will have to keep up with changing climate to avoid extinction is expected to further increase over the coming decades [76]. Because many of the traits that make species susceptible to extinction also make them vulnerable to climate change impacts, extinction risk models based on present-day knowledge (e.g., IUCN Red List) can provide insights into a species’ inherent sensitivity and capacity to adapt to a changing climate [24–26]. This information can be particularly informative for conservation when combined with a species’ likely exposure to climate change impacts [11,16,24,47,77]. We found many regions with high numbers of at-risk species that coincided with large changes in climate (Fig 3). These areas include the Amazon, South Africa, and parts of Southeast Asia, and harbored high numbers of species currently assessed as threatened on the IUCN Red List (Fig 3B, S1 Table). Regions with low numbers of currently threatened species, but projected to experience rapid climate change, include eastern North America, Arabian Peninsula, Sahara, Eastern Europe and much of dryland Australia (Fig 3B). Many latent risk species occurred in regions with high rates of projected climate change, including eastern North America, Arabian Peninsula, and Sahara (Fig 3C). These maps of climate velocity and species risk highlight areas where climate is predicted to change rapidly, but are not currently recognized as hotspots of mammalian risk by the IUCN Red List [57]. Our findings suggest that the geographic landscape of mammalian risk may therefore look much different in the future, underscoring the need to improve our ability to understand how climate change will impact species and how it will interact with other threats they are facing [77–79].

## Conclusions

Our work goes beyond simply identifying the drivers of extinction risk [e.g., [9,10,16]], by providing species-level response curves of extinction correlates, both intrinsic and extrinsic, and showing the geographic concentration of species associated with particular extinction correlates. These results help reveal where each risk factor is most likely important in driving regional extinctions and offer guidance for conservation research. The interplay of intrinsic and extrinsic variables and synergisms among threats is what ultimately determines which species are most likely to be threatened with extinction and where they occur around the globe. We use new data and analytic tools to help uncover these complex relationships. We also identify areas around the globe that are not currently recognized as regions of greatest risk for mammals but that harbor many species predisposed to high extinction risk and facing rapid climate change. The added impacts from climate change are likely to increase the susceptibility of these at-risk species to extinction, potentially manifesting as new regions of mammalian extinction risk.

## Supporting information

S1 Fig Geographic distribution of landcover types used in the random forest model, based on the ESA GlobCover 2009 project (28). (GIF)

S2 Fig Variable importance plot from the random forest model. Plot shows rank order of importance of all predictors included in the model. Because we used raw (non-imputed) data to build the conditional inference forest, variables with lots of missing data were biased against in terms of their relative importance. However, two of the top three most important trait variables (i.e., geographic range size and body size) had more missing data than any of the extrinsic variables in our model, and the top eleven predictors in our model had complete or nearly complete data. So, the relative importance among the top eleven predictors in our model does not reflect inherent biases against missing data.
(PDF)

S3 Fig Area under ROC-curve plots showing true positive rates versus false positive rates for the random forest holdout sample and full model. (PDF)

S4 Fig Cohen’s kappa distribution for the random forest holdout sample and full model. (PDF)

S1 Table Species at risk, according to the IUCN Red List and random forest model predictions. IUCN Red List Status is listed for each species: CR = Critically Endangered, DD = Data Deficient, EN = Endangered, EX = Extinct, EW = Extinct in the Wild, LC = Least Concern, NT = Near Threatened, VU = Vulnerable. Species already identified as threatened by the Red List are indicated as actually at risk (Actual), and those species not known to be threatened (LC and DD species) but predicted to be by our model are indicated as potentially at risk (Potential). Mean climate change velocity (km/year) within a species range also is indicated, which is the speed at which climate is changing based on the instantaneous horizontal velocity of temperature change between 2050–2100 (45).
(DOCX)

S2 Table Performance statistics for random forest model of mammal extinction risk. (DOCX)
