# Supplementary figures and images for: Geography of current and future global mammal extinction risk
Source: PLoS One. 2017 Nov 16;12(11):e0186934. doi: 10.1371/journal.pone.0186934 (PMC5690607; doi:10.1371/journal.pone.0186934)

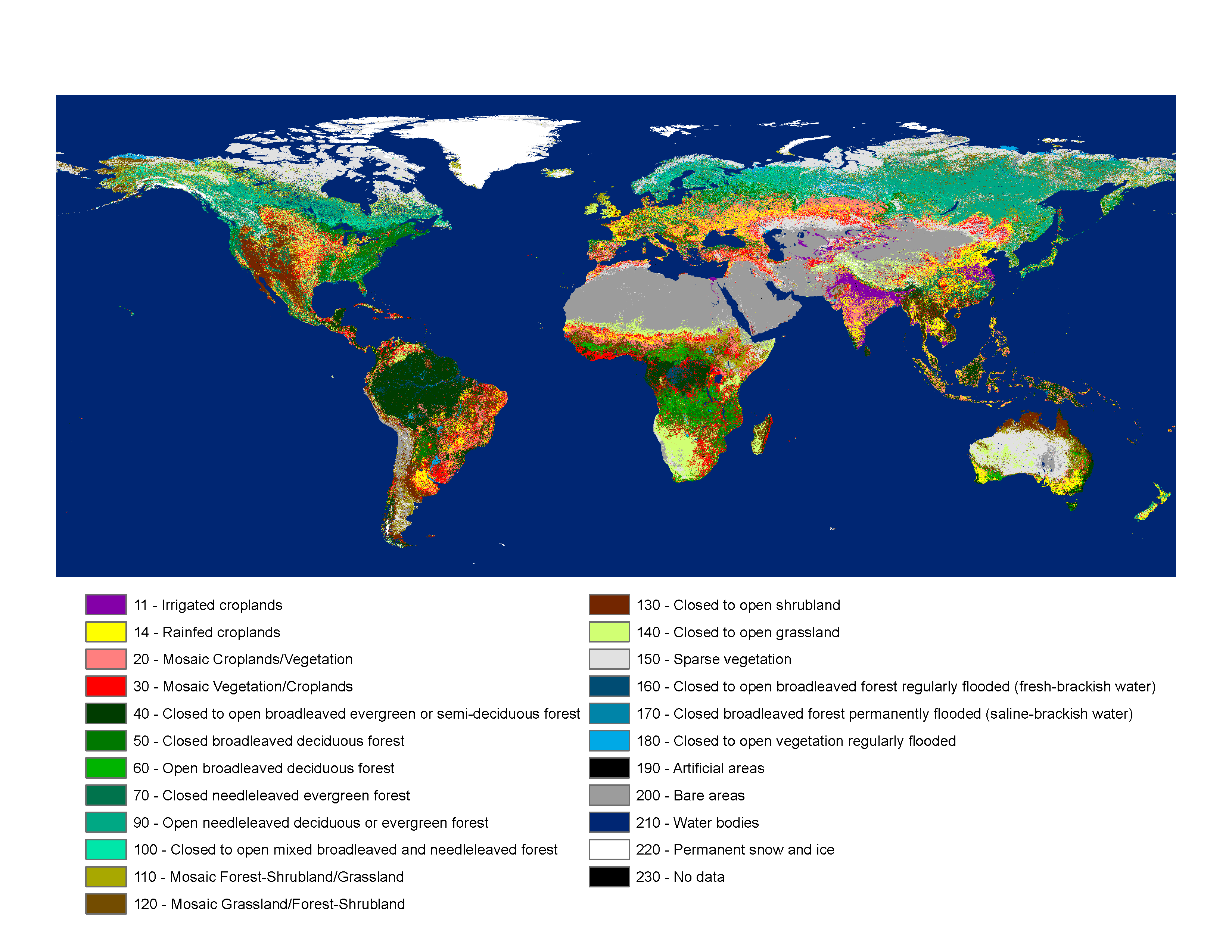

Supplement: S1 Fig — (GIF) [file pone.0186934.s001.gif]

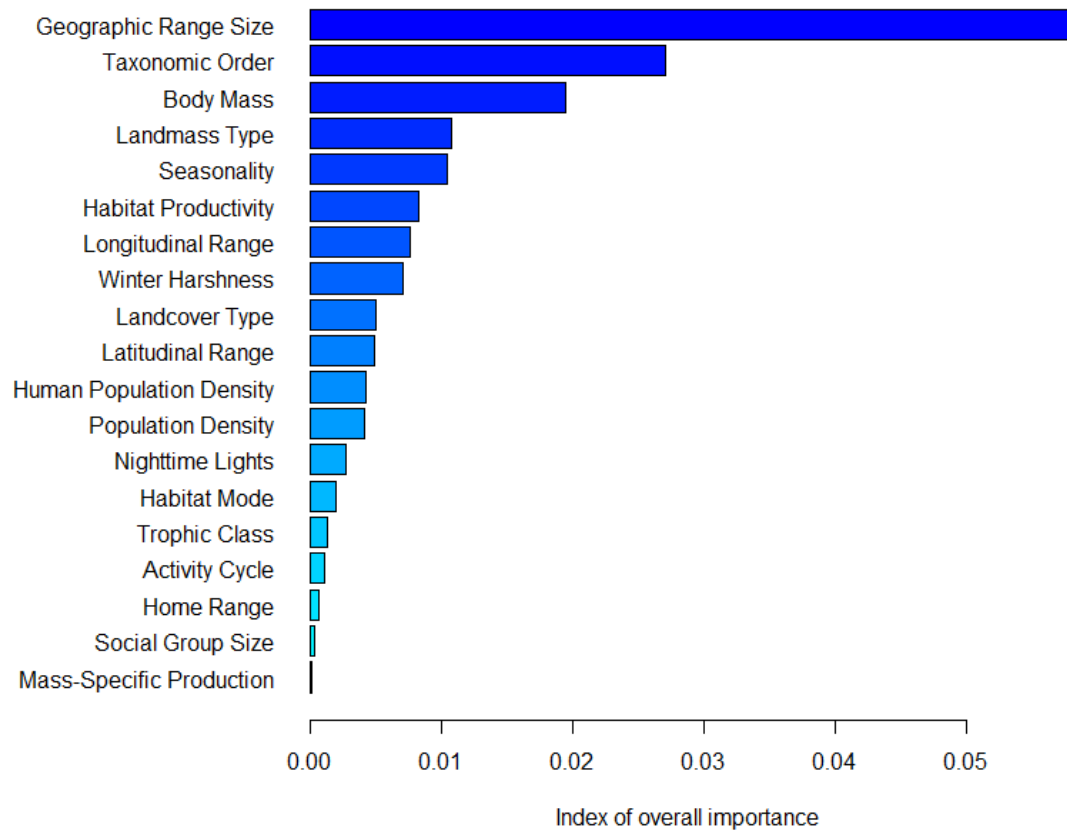

Supplement: S2 Fig — Plot shows rank order of importance of all predictors included in the model. Because we used raw (non-imputed) data to build the conditional inference forest, variables with lots of missing data were biased against in terms of their relative importance. However, two of the top three most important trait variables (i.e., geographic range size and body size) had more missing data than any of the extrinsic variables in our model, and the top eleven predictors in our model had complete or nearly complete data. So, the relative importance among the top eleven predictors in our model does not reflect inherent biases against missing data. (PDF) [file pone.0186934.s002.pdf]

**A**

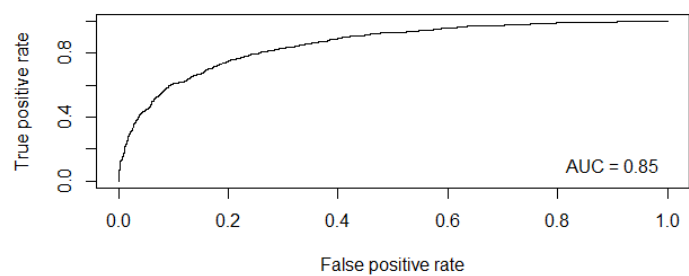

**B**

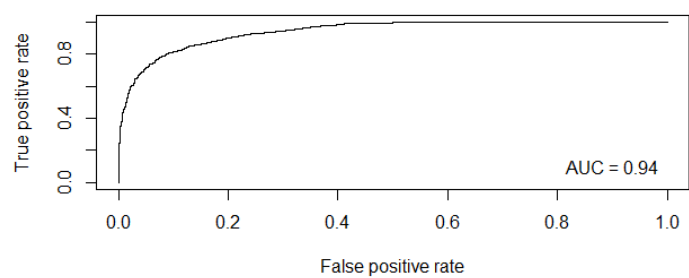

Supplement: S3 Fig — (PDF) [file pone.0186934.s003.pdf]

A

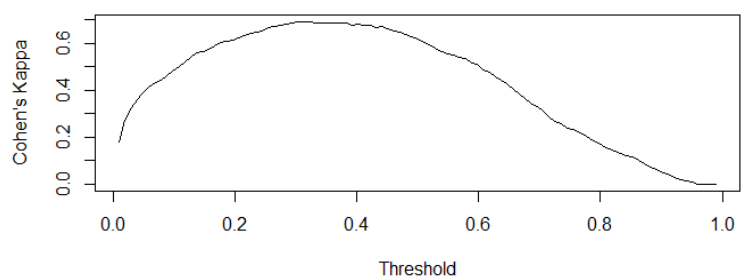

B

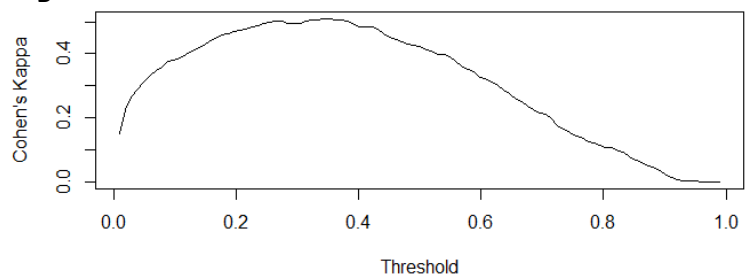

Supplement: S4 Fig — (PDF) [file pone.0186934.s004.pdf]
