# Supplementary material for: Geography of current and future global mammal extinction risk
Source: PLoS One. 2017 Nov 16;12(11):e0186934. doi: 10.1371/journal.pone.0186934 (PMC5690607; doi:10.1371/journal.pone.0186934)
Supplement: S1 Table — IUCN Red List Status is listed for each species: CR = Critically Endangered, DD = Data Deficient, EN = Endangered, EX = Extinct, EW = Extinct in the Wild, LC = Least Concern, NT = Near Threatened, VU = Vulnerable. Species already identified as threatened by the Red List are indicated as actually at risk (Actual), and those species not known to be threatened (LC and DD species) but predicted to be by our model are indicated as potentially at risk (Potential). Mean climate change velocity (km/year) within a species range also is indicated, which is the speed at which climate is changing based on the instantaneous horizontal velocity of temperature change between 2050–2100 (45). (DOCX) [file pone.0186934.s005.docx]

| **Order** | **Family** | **Species name** | **Red List Status** | **Risk** | **CC Velocity** |
| --- | --- | --- | --- | --- | --- |
| Artiodactyla | Bovidae | *Addax nasomaculatus* | CR | Actual | 1.781 |
| Artiodactyla | Bovidae | *Beatragus hunteri* | CR | Actual | 1.850 |
| Artiodactyla | Bovidae | *Bos sauveli* | CR | Actual | 0.964 |
| Artiodactyla | Bovidae | *Cephalophus adersi* | CR | Actual | 1.085 |
| Artiodactyla | Bovidae | *Nanger dama* | CR | Actual | 1.752 |
| Artiodactyla | Bovidae | *Pseudoryx nghetinhensis* | CR | Actual | 0.155 |
| Artiodactyla | Bovidae | *Saiga tatarica* | CR | Actual | 1.641 |
| Artiodactyla | Camelidae | *Camelus ferus* | CR | Actual | 0.998 |
| Artiodactyla | Suidae | *Porcula salvania* | CR | Actual | 1.491 |
| Artiodactyla | Suidae | *Sus cebifrons* | CR | Actual | 0.244 |
| Carnivora | Canidae | *Canis rufus* | CR | Actual | 1.725 |
| Carnivora | Canidae | *Pseudalopex fulvipes* | CR | Actual | 0.216 |
| Carnivora | Canidae | *Urocyon littoralis* | CR | Actual | 0.465 |
| Carnivora | Felidae | *Lynx pardinus* | CR | Actual | 0.444 |
| Carnivora | Viverridae | *Viverra civettina* | CR | Actual | 0.673 |
| Chiroptera | Emballonuridae | *Coleura seychellensis* | CR | Actual | 0.175 |
| Chiroptera | Mormoopidae | *Pteronotus paraguanensis* | CR | Actual | 0.601 |
| Chiroptera | Mystacinidae | *Mystacina robusta* | CR | Actual | 0.167 |
| Chiroptera | Natalidae | *Natalus jamaicensis* | CR | Actual | 0.157 |
| Chiroptera | Natalidae | *Natalus primus* | CR | Actual | 1.594 |
| Chiroptera | Pteropodidae | *Aproteles bulmerae* | CR | Actual | 1.278 |
| Chiroptera | Pteropodidae | *Dobsonia chapmani* | CR | Actual | 0.178 |
| Chiroptera | Pteropodidae | *Mirimiri acrodonta* | CR | Actual | 1.855 |
| Chiroptera | Pteropodidae | *Pteralopex flanneryi* | CR | Actual | 0.471 |
| Chiroptera | Pteropodidae | *Pteropus rodricensis* | CR | Actual | 0.653 |
| Chiroptera | Rhinolophidae | *Rhinolophus hilli* | CR | Actual | 1.028 |
| Chiroptera | Vespertilionidae | *Myotis hajastanicus* | CR | Actual | 0.403 |
| Chiroptera | Vespertilionidae | *Myotis yanbarensis* | CR | Actual | 0.137 |
| Chiroptera | Vespertilionidae | *Pipistrellus murrayi* | CR | Actual | 0.453 |
| Dasyuromorphia | Dasyuridae | *Sminthopsis aitkeni* | CR | Actual | 0.479 |
| Diprotodontia | Macropodidae | *Dendrolagus mbaiso* | CR | Actual | 0.054 |
| Diprotodontia | Macropodidae | *Dorcopsis atrata* | CR | Actual | 0.072 |
| Diprotodontia | Phalangeridae | *Spilocuscus rufoniger* | CR | Actual | 0.557 |
| Diprotodontia | Phalangeridae | *Spilocuscus wilsoni* | CR | Actual | 0.442 |
| Diprotodontia | Potoroidae | *Bettongia penicillata* | CR | Actual | 0.513 |
| Lagomorpha | Leporidae | *Bunolagus monticularis* | CR | Actual | 0.609 |
| Lagomorpha | Ochotonidae | *Ochotona argentata* | CR | Actual | 1.116 |
| Monotremata | Tachyglossidae | *Zaglossus bartoni* | CR | Actual | 0.122 |
| Monotremata | Tachyglossidae | *Zaglossus bruijnii* | CR | Actual | 0.537 |
| Perissodactyla | Equidae | *Equus africanus* | CR | Actual | 0.667 |
| Perissodactyla | Equidae | *Equus ferus* | CR | Actual | 0.907 |
| Perissodactyla | Rhinocerotidae | *Dicerorhinus sumatrensis* | CR | Actual | 0.221 |
| Perissodactyla | Rhinocerotidae | *Diceros bicornis* | CR | Actual | 1.130 |
| Primates | Atelidae | *Ateles fusciceps* | CR | Actual | 0.571 |
| Primates | Atelidae | *Ateles hybridus* | CR | Actual | 0.750 |
| Primates | Atelidae | *Brachyteles hypoxanthus* | CR | Actual | 0.220 |
| Primates | Atelidae | *Lagothrix lugens* | CR | Actual | 0.561 |
| Primates | Atelidae | *Oreonax flavicauda* | CR | Actual | 0.157 |
| Primates | Callitrichidae | *Saguinus oedipus* | CR | Actual | 0.997 |
| Primates | Cebidae | *Cebus flavius* | CR | Actual | 0.680 |
| Primates | Cebidae | *Cebus kaapori* | CR | Actual | 1.045 |
| Primates | Cebidae | *Cebus xanthosternos* | CR | Actual | 0.499 |
| Primates | Cercopithecidae | *Macaca nigra* | CR | Actual | 0.133 |
| Primates | Cercopithecidae | *Macaca pagensis* | CR | Actual | 0.432 |
| Primates | Cercopithecidae | *Presbytis chrysomelas* | CR | Actual | 0.789 |
| Primates | Cercopithecidae | *Procolobus pennantii* | CR | Actual | 1.581 |
| Primates | Cercopithecidae | *Procolobus preussi* | CR | Actual | 0.306 |
| Primates | Cercopithecidae | *Pygathrix cinerea* | CR | Actual | 0.176 |
| Primates | Cercopithecidae | *Rhinopithecus avunculus* | CR | Actual | 0.267 |
| Primates | Cercopithecidae | *Rungwecebus kipunji* | CR | Actual | 0.280 |
| Primates | Cercopithecidae | *Simias concolor* | CR | Actual | 0.395 |
| Primates | Cercopithecidae | *Trachypithecus delacouri* | CR | Actual | 0.667 |
| Primates | Cercopithecidae | *Trachypithecus poliocephalus* | CR | Actual | 0.511 |
| Primates | Hominidae | *Gorilla gorilla* | CR | Actual | 0.829 |
| Primates | Hominidae | *Pongo abelii* | CR | Actual | 0.320 |
| Primates | Hylobatidae | *Nomascus concolor* | CR | Actual | 0.087 |
| Primates | Hylobatidae | *Nomascus leucogenys* | CR | Actual | 0.148 |
| Primates | Hylobatidae | *Nomascus nasutus* | CR | Actual | 0.187 |
| Primates | Indridae | *Propithecus candidus* | CR | Actual | 0.184 |
| Primates | Indridae | *Propithecus perrieri* | CR | Actual | 0.233 |
| Primates | Lemuridae | *Prolemur simus* | CR | Actual | 0.347 |
| Primates | Lemuridae | *Varecia variegata* | CR | Actual | 0.279 |
| Primates | Pitheciidae | *Callicebus barbarabrownae* | CR | Actual | 0.424 |
| Primates | Pitheciidae | *Chiropotes satanas* | CR | Actual | 1.001 |
| Rodentia | Capromyidae | *Mesocapromys nanus* | CR | Actual | 1.684 |
| Rodentia | Capromyidae | *Mysateles garridoi* | CR | Actual | 0.232 |
| Rodentia | Capromyidae | *Mysateles meridionalis* | CR | Actual | 0.232 |
| Rodentia | Chinchillidae | *Chinchilla chinchilla* | CR | Actual | 0.250 |
| Rodentia | Chinchillidae | *Chinchilla lanigera* | CR | Actual | 0.068 |
| Rodentia | Cricetidae | *Habromys ixtlani* | CR | Actual | 0.051 |
| Rodentia | Cricetidae | *Habromys lepturus* | CR | Actual | 1.865 |
| Rodentia | Cricetidae | *Habromys schmidlyi* | CR | Actual | 0.129 |
| Rodentia | Cricetidae | *Microtus bavaricus* | CR | Actual | 1.888 |
| Rodentia | Cricetidae | *Peromyscus caniceps* | CR | Actual | 0.151 |
| Rodentia | Cricetidae | *Peromyscus guardia* | CR | Actual | 0.275 |
| Rodentia | Cricetidae | *Peromyscus mayensis* | CR | Actual | 0.196 |
| Rodentia | Cricetidae | *Peromyscus mekisturus* | CR | Actual | 0.305 |
| Rodentia | Cricetidae | *Tylomys tumbalensis* | CR | Actual | 0.206 |
| Rodentia | Ctenomyidae | *Ctenomys roigi* | CR | Actual | 1.865 |
| Rodentia | Ctenomyidae | *Ctenomys sociabilis* | CR | Actual | 0.165 |
| Rodentia | Dasyproctidae | *Dasyprocta mexicana* | CR | Actual | 0.895 |
| Rodentia | Echimyidae | *Phyllomys mantiqueirensis* | CR | Actual | 0.246 |
| Rodentia | Echimyidae | *Phyllomys unicolor* | CR | Actual | 0.046 |
| Rodentia | Heteromyidae | *Dipodomys gravipes* | CR | Actual | 0.448 |
| Rodentia | Muridae | *Crateromys australis* | CR | Actual | 0.320 |
| Rodentia | Muridae | *Leporillus apicalis* | CR | Actual | 1.497 |
| Rodentia | Muridae | *Melomys rubicola* | CR | Actual | 0.504 |
| Rodentia | Muridae | *Solomys ponceleti* | CR | Actual | 0.459 |
| Rodentia | Muridae | *Uromys boeadii* | CR | Actual | 0.442 |
| Rodentia | Muridae | *Uromys imperator* | CR | Actual | 2.419 |
| Rodentia | Muridae | *Uromys porculus* | CR | Actual | 2.419 |
| Rodentia | Muridae | *Zyzomys palatalis* | CR | Actual | 1.220 |
| Rodentia | Muridae | *Zyzomys pedunculatus* | CR | Actual | 0.759 |
| Rodentia | Octodontidae | *Octodon pacificus* | CR | Actual | 0.348 |
| Rodentia | Octodontidae | *Pipanacoctomys aureus* | CR | Actual | 1.489 |
| Rodentia | Sciuridae | *Biswamoyopterus biswasi* | CR | Actual | 0.310 |
| Rodentia | Sciuridae | *Marmota vancouverensis* | CR | Actual | 0.085 |
| Soricomorpha | Soricidae | *Congosorex phillipsorum* | CR | Actual | 0.203 |
| Soricomorpha | Soricidae | *Crocidura trichura* | CR | Actual | 0.302 |
| Soricomorpha | Soricidae | *Crocidura wimmeri* | CR | Actual | 0.881 |
| Afrosoricida | Chrysochloridae | *Calcochloris tytonis* | DD | Potential | 1.573 |
| Afrosoricida | Tenrecidae | *Oryzorictes tetradactylus* | DD | Potential | 0.282 |
| Artiodactyla | Bovidae | *Madoqua piacentinii* | DD | Potential | 1.114 |
| Artiodactyla | Cervidae | *Mazama nana* | DD | Potential | 0.384 |
| Artiodactyla | Cervidae | *Muntiacus feae* | DD | Potential | 0.281 |
| Artiodactyla | Cervidae | *Muntiacus gongshanensis* | DD | Potential | 0.067 |
| Artiodactyla | Cervidae | *Muntiacus montanus* | DD | Potential | 0.162 |
| Artiodactyla | Cervidae | *Muntiacus putaoensis* | DD | Potential | 0.116 |
| Artiodactyla | Cervidae | *Muntiacus rooseveltorum* | DD | Potential | 0.173 |
| Artiodactyla | Cervidae | *Muntiacus truongsonensis* | DD | Potential | 0.143 |
| Artiodactyla | Suidae | *Sus bucculentus* | DD | Potential | 0.730 |
| Artiodactyla | Tragulidae | *Tragulus versicolor* | DD | Potential | 0.387 |
| Artiodactyla | Tragulidae | *Tragulus williamsoni* | DD | Potential | 0.197 |
| Carnivora | Mustelidae | *Melogale everetti* | DD | Potential | 0.077 |
| Carnivora | Mustelidae | *Melogale orientalis* | DD | Potential | 0.244 |
| Carnivora | Mustelidae | *Mustela lutreolina* | DD | Potential | 0.120 |
| Carnivora | Procyonidae | *Bassaricyon lasius* | DD | Potential | 0.069 |
| Carnivora | Procyonidae | *Bassaricyon pauli* | DD | Potential | 0.084 |
| Carnivora | Viverridae | *Genetta poensis* | DD | Potential | 0.609 |
| Chiroptera | Emballonuridae | *Saccopteryx antioquensis* | DD | Potential | 0.084 |
| Chiroptera | Hipposideridae | *Hipposideros breviceps* | DD | Potential | 0.446 |
| Chiroptera | Hipposideridae | *Hipposideros corynophyllus* | DD | Potential | 0.049 |
| Chiroptera | Hipposideridae | *Hipposideros crumeniferus* | DD | Potential | 0.198 |
| Chiroptera | Hipposideridae | *Hipposideros edwardshilli* | DD | Potential | 0.472 |
| Chiroptera | Hipposideridae | *Hipposideros inexpectatus* | DD | Potential | 0.135 |
| Chiroptera | Hipposideridae | *Hipposideros macrobullatus* | DD | Potential | 0.195 |
| Chiroptera | Hipposideridae | *Hipposideros muscinus* | DD | Potential | 0.344 |
| Chiroptera | Molossidae | *Otomops formosus* | DD | Potential | 0.493 |
| Chiroptera | Molossidae | *Otomops papuensis* | DD | Potential | 1.047 |
| Chiroptera | Molossidae | *Otomops wroughtoni* | DD | Potential | 0.483 |
| Chiroptera | Phyllostomidae | *Lonchophylla orcesi* | DD | Potential | 0.061 |
| Chiroptera | Phyllostomidae | *Micronycteris brosseti* | DD | Potential | 1.470 |
| Chiroptera | Phyllostomidae | *Micronycteris sanborni* | DD | Potential | 0.526 |
| Chiroptera | Phyllostomidae | *Sturnira mistratensis* | DD | Potential | 0.178 |
| Chiroptera | Pteropodidae | *Eonycteris major* | DD | Potential | 0.668 |
| Chiroptera | Pteropodidae | *Nyctimene draconilla* | DD | Potential | 1.424 |
| Chiroptera | Pteropodidae | *Nyctimene malaitensis* | DD | Potential | 0.293 |
| Chiroptera | Pteropodidae | *Pteropus gilliardorum* | DD | Potential | 0.086 |
| Chiroptera | Pteropodidae | *Pteropus griseus* | DD | Potential | 0.248 |
| Chiroptera | Pteropodidae | *Pteropus intermedius* | DD | Potential | 0.939 |
| Chiroptera | Pteropodidae | *Pteropus lombocensis* | DD | Potential | 0.184 |
| Chiroptera | Rhinolophidae | *Rhinolophus adami* | DD | Potential | 0.365 |
| Chiroptera | Rhinolophidae | *Rhinolophus maendeleo* | DD | Potential | 0.278 |
| Chiroptera | Rhinolophidae | *Rhinolophus sakejiensis* | DD | Potential | 0.674 |
| Chiroptera | Thyropteridae | *Thyroptera lavali* | DD | Potential | 1.517 |
| Chiroptera | Vespertilionidae | *Eudiscopus denticulus* | DD | Potential | 0.515 |
| Chiroptera | Vespertilionidae | *Glischropus javanus* | DD | Potential | 0.145 |
| Chiroptera | Vespertilionidae | *Harpiocephalus mordax* | DD | Potential | 0.562 |
| Chiroptera | Vespertilionidae | *Harpiola grisea* | DD | Potential | 0.227 |
| Chiroptera | Vespertilionidae | *Hesperoptenus doriae* | DD | Potential | 0.418 |
| Chiroptera | Vespertilionidae | *Hesperoptenus gaskelli* | DD | Potential | 0.783 |
| Chiroptera | Vespertilionidae | *Kerivoula agnella* | DD | Potential | 0.189 |
| Chiroptera | Vespertilionidae | *Kerivoula myrella* | DD | Potential | 0.212 |
| Chiroptera | Vespertilionidae | *Lasiurus ebenus* | DD | Potential | 0.571 |
| Chiroptera | Vespertilionidae | *Myotis annamiticus* | DD | Potential | 0.169 |
| Chiroptera | Vespertilionidae | *Myotis dieteri* | DD | Potential | 0.459 |
| Chiroptera | Vespertilionidae | *Myotis hermani* | DD | Potential | 0.114 |
| Chiroptera | Vespertilionidae | *Myotis rufopictus* | DD | Potential | 0.229 |
| Chiroptera | Vespertilionidae | *Myotis stalkeri* | DD | Potential | 0.494 |
| Chiroptera | Vespertilionidae | *Nyctophilus heran* | DD | Potential | 0.207 |
| Chiroptera | Vespertilionidae | *Pipistrellus joffrei* | DD | Potential | 0.114 |
| Chiroptera | Vespertilionidae | *Pipistrellus kitcheneri* | DD | Potential | 0.730 |
| Chiroptera | Vespertilionidae | *Pipistrellus lophurus* | DD | Potential | 0.358 |
| Chiroptera | Vespertilionidae | *Pipistrellus macrotis* | DD | Potential | 1.050 |
| Chiroptera | Vespertilionidae | *Pipistrellus vordermanni* | DD | Potential | 0.863 |
| Cingulata | Dasypodidae | *Dasypus yepesi* | DD | Potential | 0.769 |
| Dasyuromorphia | Dasyuridae | *Sminthopsis archeri* | DD | Potential | 1.142 |
| Didelphimorphia | Didelphidae | *Cryptonanus guahybae* | DD | Potential | 0.802 |
| Didelphimorphia | Didelphidae | *Marmosops creightoni* | DD | Potential | 0.050 |
| Erinaceomorpha | Erinaceidae | *Hylomys megalotis* | DD | Potential | 0.386 |
| Lagomorpha | Leporidae | *Nesolagus timminsi* | DD | Potential | 0.174 |
| Lagomorpha | Leporidae | *Sylvilagus cognatus* | DD | Potential | 0.589 |
| Lagomorpha | Leporidae | *Sylvilagus dicei* | DD | Potential | 0.060 |
| Lagomorpha | Leporidae | *Sylvilagus varynaensis* | DD | Potential | 2.303 |
| Lagomorpha | Ochotonidae | *Ochotona gaoligongensis* | DD | Potential | 0.050 |
| Lagomorpha | Ochotonidae | *Ochotona muliensis* | DD | Potential | 0.052 |
| Peramelemorphia | Peramelidae | *Echymipera echinista* | DD | Potential | 1.147 |
| Primates | Aotidae | *Aotus jorgehernandezi* | DD | Potential | 0.064 |
| Primates | Aotidae | *Aotus zonalis* | DD | Potential | 0.443 |
| Primates | Callitrichidae | *Mico acariensis* | DD | Potential | 1.076 |
| Primates | Callitrichidae | *Mico chrysoleucus* | DD | Potential | 1.706 |
| Primates | Callitrichidae | *Mico emiliae* | DD | Potential | 0.958 |
| Primates | Callitrichidae | *Mico humeralifer* | DD | Potential | 1.008 |
| Primates | Callitrichidae | *Mico nigriceps* | DD | Potential | 2.015 |
| Primates | Cheirogaleidae | *Allocebus trichotis* | DD | Potential | 0.241 |
| Primates | Cheirogaleidae | *Cheirogaleus ravus* | DD | Potential | 0.819 |
| Primates | Cheirogaleidae | *Microcebus bongolavensis* | DD | Potential | 0.717 |
| Primates | Cheirogaleidae | *Microcebus jollyae* | DD | Potential | 0.632 |
| Primates | Cheirogaleidae | *Microcebus myoxinus* | DD | Potential | 0.607 |
| Primates | Cheirogaleidae | *Microcebus simmonsi* | DD | Potential | 0.255 |
| Primates | Indridae | *Avahi betsileo* | DD | Potential | 0.206 |
| Primates | Indridae | *Avahi meridionalis* | DD | Potential | 0.264 |
| Primates | Indridae | *Avahi peyrierasi* | DD | Potential | 0.245 |
| Primates | Lemuridae | *Eulemur rufus* | DD | Potential | 0.704 |
| Primates | Lepilemuridae | *Lepilemur aeeclis* | DD | Potential | 0.622 |
| Primates | Lepilemuridae | *Lepilemur ahmansonorum* | DD | Potential | 0.651 |
| Primates | Lepilemuridae | *Lepilemur betsileo* | DD | Potential | 0.224 |
| Primates | Lepilemuridae | *Lepilemur dorsalis* | DD | Potential | 0.175 |
| Primates | Lepilemuridae | *Lepilemur fleuretae* | DD | Potential | 0.331 |
| Primates | Lepilemuridae | *Lepilemur jamesorum* | DD | Potential | 0.420 |
| Primates | Lepilemuridae | *Lepilemur leucopus* | DD | Potential | 1.052 |
| Primates | Lepilemuridae | *Lepilemur microdon* | DD | Potential | 0.272 |
| Primates | Lepilemuridae | *Lepilemur milanoii* | DD | Potential | 0.302 |
| Primates | Lepilemuridae | *Lepilemur mustelinus* | DD | Potential | 0.277 |
| Primates | Lepilemuridae | *Lepilemur otto* | DD | Potential | 0.717 |
| Primates | Lepilemuridae | *Lepilemur petteri* | DD | Potential | 1.137 |
| Primates | Lepilemuridae | *Lepilemur randrianasoloi* | DD | Potential | 0.699 |
| Primates | Lepilemuridae | *Lepilemur ruficaudatus* | DD | Potential | 0.956 |
| Primates | Lepilemuridae | *Lepilemur sahamalazensis* | DD | Potential | 0.390 |
| Primates | Lepilemuridae | *Lepilemur seali* | DD | Potential | 0.254 |
| Primates | Lepilemuridae | *Lepilemur wrightae* | DD | Potential | 0.155 |
| Primates | Pitheciidae | *Callicebus stephennashi* | DD | Potential | 2.220 |
| Primates | Tarsiidae | *Tarsius lariang* | DD | Potential | 0.072 |
| Primates | Tarsiidae | *Tarsius pumilus* | DD | Potential | 0.176 |
| Rodentia | Abrocomidae | *Abrocoma famatina* | DD | Potential | 0.202 |
| Rodentia | Abrocomidae | *Abrocoma uspallata* | DD | Potential | 0.073 |
| Rodentia | Abrocomidae | *Abrocoma vaccarum* | DD | Potential | 0.026 |
| Rodentia | Caviidae | *Kerodon acrobata* | DD | Potential | 0.801 |
| Rodentia | Cricetidae | *Akodon lindberghi* | DD | Potential | 0.276 |
| Rodentia | Cricetidae | *Akodon oenos* | DD | Potential | 1.786 |
| Rodentia | Cricetidae | *Akodon pervalens* | DD | Potential | 0.165 |
| Rodentia | Cricetidae | *Alticola albicaudus* | DD | Potential | 0.075 |
| Rodentia | Cricetidae | *Brucepattersonius griserufescens* | DD | Potential | 0.149 |
| Rodentia | Cricetidae | *Chelemys delfini* | DD | Potential | 0.200 |
| Rodentia | Cricetidae | *Chibchanomys orcesi* | DD | Potential | 0.130 |
| Rodentia | Cricetidae | *Dicrostonyx unalascensis* | DD | Potential | 0.099 |
| Rodentia | Cricetidae | *Euneomys fossor* | DD | Potential | 2.001 |
| Rodentia | Cricetidae | *Ichthyomys tweedii* | DD | Potential | 0.103 |
| Rodentia | Cricetidae | *Oecomys cleberi* | DD | Potential | 0.426 |
| Rodentia | Cricetidae | *Oligoryzomys rupestris* | DD | Potential | 0.906 |
| Rodentia | Cricetidae | *Phyllotis osgoodi* | DD | Potential | 0.156 |
| Rodentia | Cricetidae | *Proedromys liangshanensis* | DD | Potential | 0.058 |
| Rodentia | Cricetidae | *Rhipidomys cariri* | DD | Potential | 0.434 |
| Rodentia | Cricetidae | *Rhipidomys ochrogaster* | DD | Potential | 0.096 |
| Rodentia | Cricetidae | *Thomasomys rosalinda* | DD | Potential | 0.058 |
| Rodentia | Cricetidae | *Tylomys panamensis* | DD | Potential | 0.219 |
| Rodentia | Ctenomyidae | *Ctenomys tucumanus* | DD | Potential | 1.776 |
| Rodentia | Echimyidae | *Proechimys oconnelli* | DD | Potential | 1.406 |
| Rodentia | Echimyidae | *Proechimys trinitatus* | DD | Potential | 0.908 |
| Rodentia | Muridae | *Acomys minous* | DD | Potential | 0.132 |
| Rodentia | Muridae | *Acomys nesiotes* | DD | Potential | 0.362 |
| Rodentia | Muridae | *Aethomys silindensis* | DD | Potential | 0.318 |
| Rodentia | Muridae | *Anonymomys mindorensis* | DD | Potential | 0.242 |
| Rodentia | Muridae | *Apomys littoralis* | DD | Potential | 0.393 |
| Rodentia | Muridae | *Carpomys melanurus* | DD | Potential | 0.098 |
| Rodentia | Muridae | *Chiropodomys muroides* | DD | Potential | 0.167 |
| Rodentia | Muridae | *Crateromys paulus* | DD | Potential | 0.668 |
| Rodentia | Muridae | *Crunomys celebensis* | DD | Potential | 0.139 |
| Rodentia | Muridae | *Gerbillus lowei* | DD | Potential | 0.314 |
| Rodentia | Muridae | *Gerbillus nancillus* | DD | Potential | 1.889 |
| Rodentia | Muridae | *Gerbillus principulus* | DD | Potential | 0.507 |
| Rodentia | Muridae | *Haeromys margarettae* | DD | Potential | 0.304 |
| Rodentia | Muridae | *Lemniscomys hoogstraali* | DD | Potential | 2.487 |
| Rodentia | Muridae | *Margaretamys parvus* | DD | Potential | 0.106 |
| Rodentia | Muridae | *Maxomys baeodon* | DD | Potential | 0.326 |
| Rodentia | Muridae | *Melasmothrix naso* | DD | Potential | 0.106 |
| Rodentia | Muridae | *Melomys bougainville* | DD | Potential | 0.459 |
| Rodentia | Muridae | *Melomys fulgens* | DD | Potential | 0.063 |
| Rodentia | Muridae | *Melomys howi* | DD | Potential | 0.559 |
| Rodentia | Muridae | *Microhydromys musseri* | DD | Potential | 0.147 |
| Rodentia | Muridae | *Mus crociduroides* | DD | Potential | 0.116 |
| Rodentia | Muridae | *Mus fragilicauda* | DD | Potential | 0.241 |
| Rodentia | Muridae | *Mus goundae* | DD | Potential | 0.552 |
| Rodentia | Muridae | *Niviventer hinpoon* | DD | Potential | 0.532 |
| Rodentia | Muridae | *Palawanomys furvus* | DD | Potential | 0.284 |
| Rodentia | Muridae | *Pelomys isseli* | DD | Potential | 1.958 |
| Rodentia | Muridae | *Pithecheir parvus* | DD | Potential | 0.527 |
| Rodentia | Muridae | *Praomys minor* | DD | Potential | 0.461 |
| Rodentia | Muridae | *Pseudohydromys germani* | DD | Potential | 0.087 |
| Rodentia | Muridae | *Pseudomys pilligaensis* | DD | Potential | 0.669 |
| Rodentia | Muridae | *Rattus bontanus* | DD | Potential | 0.117 |
| Rodentia | Muridae | *Rattus enganus* | DD | Potential | 0.446 |
| Rodentia | Muridae | *Rattus mindorensis* | DD | Potential | 0.243 |
| Rodentia | Muridae | *Rhynchomys tapulao* | DD | Potential | 0.178 |
| Rodentia | Muridae | *Saxatilomys paulinae* | DD | Potential | 0.502 |
| Rodentia | Muridae | *Stenocephalemys ruppi* | DD | Potential | 0.193 |
| Rodentia | Muridae | *Taeromys hamatus* | DD | Potential | 0.112 |
| Rodentia | Nesomyidae | *Megadendromus nikolausi* | DD | Potential | 0.209 |
| Rodentia | Octodontidae | *Aconaemys sagei* | DD | Potential | 0.220 |
| Rodentia | Sciuridae | *Eupetaurus cinereus* | DD | Potential | 0.036 |
| Rodentia | Sciuridae | *Hylopetes winstoni* | DD | Potential | 0.074 |
| Rodentia | Sciuridae | *Prosciurillus abstrusus* | DD | Potential | 0.069 |
| Rodentia | Sciuridae | *Prosciurillus weberi* | DD | Potential | 0.278 |
| Soricomorpha | Soricidae | *Chodsigoa caovansunga* | DD | Potential | 0.077 |
| Soricomorpha | Soricidae | *Congosorex polli* | DD | Potential | 0.729 |
| Soricomorpha | Soricidae | *Crocidura fischeri* | DD | Potential | 0.564 |
| Soricomorpha | Soricidae | *Crocidura floweri* | DD | Potential | 1.516 |
| Soricomorpha | Soricidae | *Crocidura hilliana* | DD | Potential | 0.545 |
| Soricomorpha | Soricidae | *Crocidura selina* | DD | Potential | 0.736 |
| Soricomorpha | Soricidae | *Crocidura tenuis* | DD | Potential | 0.203 |
| Soricomorpha | Soricidae | *Crocidura vosmaeri* | DD | Potential | 1.188 |
| Soricomorpha | Soricidae | *Crocidura zaphiri* | DD | Potential | 0.390 |
| Soricomorpha | Soricidae | *Cryptotis peregrina* | DD | Potential | 0.136 |
| Soricomorpha | Soricidae | *Cryptotis peruviensis* | DD | Potential | 0.052 |
| Soricomorpha | Soricidae | *Sorex alaskanus* | DD | Potential | 0.441 |
| Soricomorpha | Soricidae | *Sorex arunchi* | DD | Potential | 0.433 |
| Soricomorpha | Soricidae | *Sorex leucogaster* | DD | Potential | 0.169 |
| Soricomorpha | Soricidae | *Sorex neomexicanus* | DD | Potential | 0.570 |
| Soricomorpha | Soricidae | *Sylvisorex pluvialis* | DD | Potential | 0.545 |
| Soricomorpha | Talpidae | *Talpa davidiana* | DD | Potential | 0.451 |
| Afrosoricida | Chrysochloridae | *Amblysomus marleyi* | EN | Actual | 0.505 |
| Afrosoricida | Chrysochloridae | *Chrysospalax trevelyani* | EN | Actual | 0.219 |
| Afrosoricida | Chrysochloridae | *Cryptochloris zyli* | EN | Actual | 0.599 |
| Afrosoricida | Chrysochloridae | *Neamblysomus gunningi* | EN | Actual | 0.288 |
| Afrosoricida | Tenrecidae | *Microgale jenkinsae* | EN | Actual | 1.170 |
| Afrosoricida | Tenrecidae | *Micropotamogale lamottei* | EN | Actual | 0.623 |
| Artiodactyla | Bovidae | *Arabitragus jayakari* | EN | Actual | 0.283 |
| Artiodactyla | Bovidae | *Bos javanicus* | EN | Actual | 0.577 |
| Artiodactyla | Bovidae | *Bubalus arnee* | EN | Actual | 1.093 |
| Artiodactyla | Bovidae | *Bubalus depressicornis* | EN | Actual | 0.219 |
| Artiodactyla | Bovidae | *Bubalus quarlesi* | EN | Actual | 0.191 |
| Artiodactyla | Bovidae | *Capra caucasica* | EN | Actual | 0.043 |
| Artiodactyla | Bovidae | *Capra falconeri* | EN | Actual | 0.375 |
| Artiodactyla | Bovidae | *Capra walie* | EN | Actual | 0.056 |
| Artiodactyla | Bovidae | *Cephalophus jentinki* | EN | Actual | 0.948 |
| Artiodactyla | Bovidae | *Cephalophus spadix* | EN | Actual | 0.285 |
| Artiodactyla | Bovidae | *Gazella cuvieri* | EN | Actual | 0.754 |
| Artiodactyla | Bovidae | *Gazella leptoceros* | EN | Actual | 1.553 |
| Artiodactyla | Bovidae | *Gazella spekei* | EN | Actual | 1.135 |
| Artiodactyla | Bovidae | *Kobus megaceros* | EN | Actual | 2.415 |
| Artiodactyla | Bovidae | *Nilgiritragus hylocrius* | EN | Actual | 0.452 |
| Artiodactyla | Bovidae | *Oryx leucoryx* | EN | Actual | 1.495 |
| Artiodactyla | Bovidae | *Pantholops hodgsonii* | EN | Actual | 0.446 |
| Artiodactyla | Bovidae | *Procapra przewalskii* | EN | Actual | 0.243 |
| Artiodactyla | Bovidae | *Pseudois schaeferi* | EN | Actual | 0.066 |
| Artiodactyla | Bovidae | *Tragelaphus buxtoni* | EN | Actual | 0.143 |
| Artiodactyla | Cervidae | *Axis calamianensis* | EN | Actual | 0.433 |
| Artiodactyla | Cervidae | *Axis porcinus* | EN | Actual | 1.603 |
| Artiodactyla | Cervidae | *Hippocamelus bisulcus* | EN | Actual | 0.101 |
| Artiodactyla | Cervidae | *Muntiacus vuquangensis* | EN | Actual | 0.227 |
| Artiodactyla | Cervidae | *Rucervus eldii* | EN | Actual | 0.897 |
| Artiodactyla | Cervidae | *Rusa alfredi* | EN | Actual | 0.234 |
| Artiodactyla | Hippopotamidae | *Choeropsis liberiensis* | EN | Actual | 0.876 |
| Artiodactyla | Moschidae | *Moschus anhuiensis* | EN | Actual | 0.228 |
| Artiodactyla | Moschidae | *Moschus berezovskii* | EN | Actual | 0.173 |
| Artiodactyla | Moschidae | *Moschus chrysogaster* | EN | Actual | 0.267 |
| Artiodactyla | Moschidae | *Moschus cupreus* | EN | Actual | 0.046 |
| Artiodactyla | Moschidae | *Moschus fuscus* | EN | Actual | 0.082 |
| Artiodactyla | Moschidae | *Moschus leucogaster* | EN | Actual | 0.073 |
| Artiodactyla | Suidae | *Babyrousa togeanensis* | EN | Actual | 0.052 |
| Artiodactyla | Suidae | *Sus oliveri* | EN | Actual | 0.205 |
| Artiodactyla | Suidae | *Sus verrucosus* | EN | Actual | 0.221 |
| Artiodactyla | Tayassuidae | *Catagonus wagneri* | EN | Actual | 2.318 |
| Carnivora | Canidae | *Canis simensis* | EN | Actual | 0.151 |
| Carnivora | Canidae | *Cuon alpinus* | EN | Actual | 0.527 |
| Carnivora | Canidae | *Lycaon pictus* | EN | Actual | 1.737 |
| Carnivora | Eupleridae | *Galidictis grandidieri* | EN | Actual | 0.595 |
| Carnivora | Felidae | *Leopardus jacobita* | EN | Actual | 0.269 |
| Carnivora | Felidae | *Panthera tigris* | EN | Actual | 0.451 |
| Carnivora | Felidae | *Panthera uncia* | EN | Actual | 0.245 |
| Carnivora | Felidae | *Pardofelis badia* | EN | Actual | 0.231 |
| Carnivora | Felidae | *Prionailurus planiceps* | EN | Actual | 0.850 |
| Carnivora | Felidae | *Prionailurus viverrinus* | EN | Actual | 0.467 |
| Carnivora | Mustelidae | *Enhydra lutris* | EN | Actual | 82.812 |
| Carnivora | Mustelidae | *Lontra provocax* | EN | Actual | 0.378 |
| Carnivora | Mustelidae | *Lutra sumatrana* | EN | Actual | 0.718 |
| Carnivora | Mustelidae | *Mustela lutreola* | EN | Actual | 1.231 |
| Carnivora | Mustelidae | *Mustela nigripes* | EN | Actual | 0.804 |
| Carnivora | Mustelidae | *Pteronura brasiliensis* | EN | Actual | 1.271 |
| Carnivora | Ursidae | *Ailuropoda melanoleuca* | EN | Actual | 0.099 |
| Carnivora | Viverridae | *Cynogale bennettii* | EN | Actual | 0.826 |
| Chiroptera | Emballonuridae | *Balantiopteryx infusca* | EN | Actual | 0.308 |
| Chiroptera | Emballonuridae | *Emballonura semicaudata* | EN | Actual | 2940.530 |
| Chiroptera | Furipteridae | *Amorphochilus schnablii* | EN | Actual | 0.692 |
| Chiroptera | Hipposideridae | *Hipposideros halophyllus* | EN | Actual | 0.934 |
| Chiroptera | Hipposideridae | *Hipposideros orbiculus* | EN | Actual | 0.637 |
| Chiroptera | Molossidae | *Tadarida bregullae* | EN | Actual | 0.129 |
| Chiroptera | Phyllostomidae | *Leptonycteris nivalis* | EN | Actual | 0.482 |
| Chiroptera | Phyllostomidae | *Lonchorhina fernandezi* | EN | Actual | 0.755 |
| Chiroptera | Phyllostomidae | *Lonchorhina marinkellei* | EN | Actual | 1.593 |
| Chiroptera | Phyllostomidae | *Platyrrhinus chocoensis* | EN | Actual | 0.463 |
| Chiroptera | Phyllostomidae | *Sturnira nana* | EN | Actual | 0.336 |
| Chiroptera | Pteropodidae | *Acerodon humilis* | EN | Actual | 0.261 |
| Chiroptera | Pteropodidae | *Acerodon jubatus* | EN | Actual | 0.298 |
| Chiroptera | Pteropodidae | *Latidens salimalii* | EN | Actual | 0.349 |
| Chiroptera | Pteropodidae | *Myonycteris brachycephala* | EN | Actual | 0.909 |
| Chiroptera | Pteropodidae | *Neopteryx frosti* | EN | Actual | 0.152 |
| Chiroptera | Pteropodidae | *Nyctimene rabori* | EN | Actual | 0.254 |
| Chiroptera | Pteropodidae | *Pteralopex anceps* | EN | Actual | 0.459 |
| Chiroptera | Pteropodidae | *Pteralopex atrata* | EN | Actual | 2.419 |
| Chiroptera | Pteropodidae | *Pteralopex taki* | EN | Actual | 0.395 |
| Chiroptera | Pteropodidae | *Pteropus capistratus* | EN | Actual | 0.223 |
| Chiroptera | Pteropodidae | *Pteropus cognatus* | EN | Actual | 0.211 |
| Chiroptera | Pteropodidae | *Pteropus fundatus* | EN | Actual | 0.601 |
| Chiroptera | Pteropodidae | *Pteropus livingstonii* | EN | Actual | 0.167 |
| Chiroptera | Pteropodidae | *Pteropus mariannus* | EN | Actual | 0.534 |
| Chiroptera | Pteropodidae | *Pteropus melanopogon* | EN | Actual | 0.175 |
| Chiroptera | Pteropodidae | *Pteropus nitendiensis* | EN | Actual | 0.106 |
| Chiroptera | Pteropodidae | *Pteropus pohlei* | EN | Actual | 0.102 |
| Chiroptera | Rhinolophidae | *Rhinolophus maclaudi* | EN | Actual | 0.288 |
| Chiroptera | Rhinolophidae | *Rhinolophus madurensis* | EN | Actual | 0.701 |
| Chiroptera | Rhinolophidae | *Rhinolophus ziama* | EN | Actual | 0.848 |
| Chiroptera | Vespertilionidae | *Eptesicus japonensis* | EN | Actual | 0.120 |
| Chiroptera | Vespertilionidae | *Eptesicus malagasyensis* | EN | Actual | 0.285 |
| Chiroptera | Vespertilionidae | *Kerivoula africana* | EN | Actual | 0.680 |
| Chiroptera | Vespertilionidae | *Myotis peninsularis* | EN | Actual | 0.238 |
| Chiroptera | Vespertilionidae | *Myotis planiceps* | EN | Actual | 0.468 |
| Chiroptera | Vespertilionidae | *Myotis pruinosus* | EN | Actual | 0.376 |
| Chiroptera | Vespertilionidae | *Myotis sodalis* | EN | Actual | 1.196 |
| Chiroptera | Vespertilionidae | *Pipistrellus endoi* | EN | Actual | 0.194 |
| Chiroptera | Vespertilionidae | *Pipistrellus maderensis* | EN | Actual | 0.072 |
| Chiroptera | Vespertilionidae | *Plecotus teneriffae* | EN | Actual | 0.072 |
| Chiroptera | Vespertilionidae | *Rhogeessa genowaysi* | EN | Actual | 0.578 |
| Dasyuromorphia | Dasyuridae | *Dasyurus hallucatus* | EN | Actual | 0.917 |
| Dasyuromorphia | Dasyuridae | *Parantechinus apicalis* | EN | Actual | 0.538 |
| Dasyuromorphia | Dasyuridae | *Pseudantechinus mimulus* | EN | Actual | 1.127 |
| Dasyuromorphia | Dasyuridae | *Sarcophilus harrisii* | EN | Actual | 0.157 |
| Dasyuromorphia | Dasyuridae | *Sminthopsis psammophila* | EN | Actual | 1.220 |
| Dasyuromorphia | Myrmecobiidae | *Myrmecobius fasciatus* | EN | Actual | 0.635 |
| Diprotodontia | Macropodidae | *Dendrolagus goodfellowi* | EN | Actual | 0.107 |
| Diprotodontia | Macropodidae | *Dendrolagus matschiei* | EN | Actual | 0.108 |
| Diprotodontia | Macropodidae | *Dendrolagus notatus* | EN | Actual | 0.148 |
| Diprotodontia | Macropodidae | *Onychogalea fraenata* | EN | Actual | 1.041 |
| Diprotodontia | Macropodidae | *Petrogale persephone* | EN | Actual | 0.444 |
| Diprotodontia | Petauridae | *Gymnobelideus leadbeateri* | EN | Actual | 0.093 |
| Diprotodontia | Petauridae | *Petaurus gracilis* | EN | Actual | 0.526 |
| Diprotodontia | Phalangeridae | *Phalanger alexandrae* | EN | Actual | 0.415 |
| Diprotodontia | Potoroidae | *Potorous longipes* | EN | Actual | 0.105 |
| Erinaceomorpha | Erinaceidae | *Neohylomys hainanensis* | EN | Actual | 0.188 |
| Erinaceomorpha | Erinaceidae | *Podogymnura aureospinula* | EN | Actual | 0.134 |
| Lagomorpha | Leporidae | *Caprolagus hispidus* | EN | Actual | 1.606 |
| Lagomorpha | Leporidae | *Pentalagus furnessi* | EN | Actual | 0.076 |
| Lagomorpha | Leporidae | *Sylvilagus graysoni* | EN | Actual | 0.415 |
| Lagomorpha | Leporidae | *Sylvilagus robustus* | EN | Actual | 0.587 |
| Lagomorpha | Ochotonidae | *Ochotona hoffmanni* | EN | Actual | 0.333 |
| Lagomorpha | Ochotonidae | *Ochotona iliensis* | EN | Actual | 0.082 |
| Lagomorpha | Ochotonidae | *Ochotona koslowi* | EN | Actual | 0.650 |
| Macroscelidea | Macroscelididae | *Rhynchocyon chrysopygus* | EN | Actual | 1.128 |
| Peramelemorphia | Peramelidae | *Peroryctes broadbenti* | EN | Actual | 0.391 |
| Perissodactyla | Equidae | *Equus grevyi* | EN | Actual | 0.733 |
| Perissodactyla | Equidae | *Equus hemionus* | EN | Actual | 0.994 |
| Perissodactyla | Tapiridae | *Tapirus bairdii* | EN | Actual | 0.665 |
| Perissodactyla | Tapiridae | *Tapirus indicus* | EN | Actual | 0.568 |
| Perissodactyla | Tapiridae | *Tapirus pinchaque* | EN | Actual | 0.067 |
| Pholidota | Manidae | *Manis javanica* | EN | Actual | 0.635 |
| Pholidota | Manidae | *Manis pentadactyla* | EN | Actual | 0.323 |
| Pilosa | Bradypodidae | *Bradypus torquatus* | EN | Actual | 0.498 |
| Primates | Atelidae | *Alouatta pigra* | EN | Actual | 1.083 |
| Primates | Atelidae | *Alouatta ululata* | EN | Actual | 0.779 |
| Primates | Atelidae | *Ateles belzebuth* | EN | Actual | 1.385 |
| Primates | Atelidae | *Ateles chamek* | EN | Actual | 1.602 |
| Primates | Atelidae | *Ateles geoffroyi* | EN | Actual | 0.694 |
| Primates | Atelidae | *Ateles marginatus* | EN | Actual | 0.942 |
| Primates | Atelidae | *Brachyteles arachnoides* | EN | Actual | 0.300 |
| Primates | Atelidae | *Lagothrix cana* | EN | Actual | 1.435 |
| Primates | Callitrichidae | *Callithrix flaviceps* | EN | Actual | 0.153 |
| Primates | Callitrichidae | *Leontopithecus chrysomelas* | EN | Actual | 0.323 |
| Primates | Callitrichidae | *Leontopithecus chrysopygus* | EN | Actual | 0.426 |
| Primates | Callitrichidae | *Leontopithecus rosalia* | EN | Actual | 0.782 |
| Primates | Callitrichidae | *Saguinus bicolor* | EN | Actual | 1.175 |
| Primates | Callitrichidae | *Saguinus leucopus* | EN | Actual | 0.524 |
| Primates | Cebidae | *Cebus robustus* | EN | Actual | 0.364 |
| Primates | Cercopithecidae | *Cercocebus sanjei* | EN | Actual | 0.504 |
| Primates | Cercopithecidae | *Cercopithecus preussi* | EN | Actual | 0.303 |
| Primates | Cercopithecidae | *Macaca maura* | EN | Actual | 0.326 |
| Primates | Cercopithecidae | *Macaca munzala* | EN | Actual | 0.040 |
| Primates | Cercopithecidae | *Macaca silenus* | EN | Actual | 0.341 |
| Primates | Cercopithecidae | *Macaca sinica* | EN | Actual | 0.687 |
| Primates | Cercopithecidae | *Macaca sylvanus* | EN | Actual | 0.242 |
| Primates | Cercopithecidae | *Mandrillus leucophaeus* | EN | Actual | 0.430 |
| Primates | Cercopithecidae | *Nasalis larvatus* | EN | Actual | 0.800 |
| Primates | Cercopithecidae | *Presbytis comata* | EN | Actual | 0.243 |
| Primates | Cercopithecidae | *Presbytis melalophos* | EN | Actual | 0.800 |
| Primates | Cercopithecidae | *Presbytis potenziani* | EN | Actual | 0.395 |
| Primates | Cercopithecidae | *Procolobus badius* | EN | Actual | 22.644 |
| Primates | Cercopithecidae | *Procolobus gordonorum* | EN | Actual | 0.533 |
| Primates | Cercopithecidae | *Procolobus kirkii* | EN | Actual | 1.075 |
| Primates | Cercopithecidae | *Pygathrix nemaeus* | EN | Actual | 0.301 |
| Primates | Cercopithecidae | *Pygathrix nigripes* | EN | Actual | 0.450 |
| Primates | Cercopithecidae | *Rhinopithecus bieti* | EN | Actual | 0.051 |
| Primates | Cercopithecidae | *Rhinopithecus brelichi* | EN | Actual | 0.103 |
| Primates | Cercopithecidae | *Rhinopithecus roxellana* | EN | Actual | 0.204 |
| Primates | Cercopithecidae | *Semnopithecus ajax* | EN | Actual | 0.076 |
| Primates | Cercopithecidae | *Trachypithecus francoisi* | EN | Actual | 0.223 |
| Primates | Cercopithecidae | *Trachypithecus geei* | EN | Actual | 1.106 |
| Primates | Cercopithecidae | *Trachypithecus germaini* | EN | Actual | 0.845 |
| Primates | Cercopithecidae | *Trachypithecus hatinhensis* | EN | Actual | 0.333 |
| Primates | Cercopithecidae | *Trachypithecus phayrei* | EN | Actual | 0.578 |
| Primates | Cercopithecidae | *Trachypithecus shortridgei* | EN | Actual | 0.038 |
| Primates | Cercopithecidae | *Trachypithecus vetulus* | EN | Actual | 0.676 |
| Primates | Cheirogaleidae | *Microcebus berthae* | EN | Actual | 0.944 |
| Primates | Cheirogaleidae | *Microcebus ravelobensis* | EN | Actual | 0.819 |
| Primates | Cheirogaleidae | *Microcebus sambiranensis* | EN | Actual | 0.208 |
| Primates | Cheirogaleidae | *Microcebus tavaratra* | EN | Actual | 0.305 |
| Primates | Hominidae | *Gorilla beringei* | EN | Actual | 0.406 |
| Primates | Hominidae | *Pan paniscus* | EN | Actual | 1.005 |
| Primates | Hominidae | *Pan troglodytes* | EN | Actual | 1.329 |
| Primates | Hominidae | *Pongo pygmaeus* | EN | Actual | 0.764 |
| Primates | Hylobatidae | *Hoolock hoolock* | EN | Actual | 0.647 |
| Primates | Hylobatidae | *Hylobates agilis* | EN | Actual | 0.875 |
| Primates | Hylobatidae | *Hylobates albibarbis* | EN | Actual | 0.927 |
| Primates | Hylobatidae | *Hylobates klossii* | EN | Actual | 0.395 |
| Primates | Hylobatidae | *Hylobates lar* | EN | Actual | 0.552 |
| Primates | Hylobatidae | *Hylobates moloch* | EN | Actual | 0.220 |
| Primates | Hylobatidae | *Hylobates muelleri* | EN | Actual | 0.554 |
| Primates | Hylobatidae | *Hylobates pileatus* | EN | Actual | 0.920 |
| Primates | Hylobatidae | *Nomascus gabriellae* | EN | Actual | 0.477 |
| Primates | Hylobatidae | *Nomascus siki* | EN | Actual | 0.236 |
| Primates | Hylobatidae | *Symphalangus syndactylus* | EN | Actual | 0.490 |
| Primates | Indridae | *Avahi cleesei* | EN | Actual | 0.443 |
| Primates | Indridae | *Avahi occidentalis* | EN | Actual | 0.875 |
| Primates | Indridae | *Indri indri* | EN | Actual | 0.256 |
| Primates | Indridae | *Propithecus coquereli* | EN | Actual | 0.760 |
| Primates | Indridae | *Propithecus coronatus* | EN | Actual | 0.537 |
| Primates | Indridae | *Propithecus diadema* | EN | Actual | 0.282 |
| Primates | Indridae | *Propithecus edwardsi* | EN | Actual | 0.247 |
| Primates | Indridae | *Propithecus tattersalli* | EN | Actual | 0.302 |
| Primates | Lemuridae | *Eulemur cinereiceps* | EN | Actual | 0.215 |
| Primates | Lemuridae | *Eulemur sanfordi* | EN | Actual | 0.293 |
| Primates | Lemuridae | *Hapalemur aureus* | EN | Actual | 0.219 |
| Primates | Lemuridae | *Varecia rubra* | EN | Actual | 0.300 |
| Primates | Lepilemuridae | *Lepilemur ankaranensis* | EN | Actual | 0.252 |
| Primates | Loridae | *Loris tardigradus* | EN | Actual | 0.400 |
| Primates | Loridae | *Nycticebus javanicus* | EN | Actual | 0.463 |
| Primates | Pitheciidae | *Callicebus coimbrai* | EN | Actual | 0.421 |
| Primates | Pitheciidae | *Callicebus modestus* | EN | Actual | 2.187 |
| Primates | Pitheciidae | *Callicebus oenanthe* | EN | Actual | 0.402 |
| Primates | Pitheciidae | *Chiropotes albinasus* | EN | Actual | 1.050 |
| Primates | Pitheciidae | *Chiropotes utahickae* | EN | Actual | 1.048 |
| Primates | Tarsiidae | *Tarsius pelengensis* | EN | Actual | 0.235 |
| Proboscidea | Elephantidae | *Elephas maximus* | EN | Actual | 0.633 |
| Rodentia | Capromyidae | *Mesocapromys angelcabrerai* | EN | Actual | 0.047 |
| Rodentia | Capromyidae | *Mysateles gundlachi* | EN | Actual | 1.760 |
| Rodentia | Capromyidae | *Plagiodontia aedium* | EN | Actual | 0.152 |
| Rodentia | Cricetidae | *Euryoryzomys lamia* | EN | Actual | 0.412 |
| Rodentia | Cricetidae | *Habromys simulatus* | EN | Actual | 0.222 |
| Rodentia | Cricetidae | *Kunsia fronto* | EN | Actual | 1.890 |
| Rodentia | Cricetidae | *Megadontomys cryophilus* | EN | Actual | 0.092 |
| Rodentia | Cricetidae | *Megadontomys nelsoni* | EN | Actual | 0.297 |
| Rodentia | Cricetidae | *Microtus oaxacensis* | EN | Actual | 1.639 |
| Rodentia | Cricetidae | *Microtus umbrosus* | EN | Actual | 0.071 |
| Rodentia | Cricetidae | *Mindomys hammondi* | EN | Actual | 0.070 |
| Rodentia | Cricetidae | *Nelsonia goldmani* | EN | Actual | 0.117 |
| Rodentia | Cricetidae | *Neotoma angustapalata* | EN | Actual | 0.312 |
| Rodentia | Cricetidae | *Neusticomys mussoi* | EN | Actual | 0.055 |
| Rodentia | Cricetidae | *Oxymycterus hucucha* | EN | Actual | 0.118 |
| Rodentia | Cricetidae | *Oxymycterus josei* | EN | Actual | 0.857 |
| Rodentia | Cricetidae | *Peromyscus melanocarpus* | EN | Actual | 0.067 |
| Rodentia | Cricetidae | *Peromyscus melanurus* | EN | Actual | 0.123 |
| Rodentia | Cricetidae | *Peromyscus ochraventer* | EN | Actual | 0.358 |
| Rodentia | Cricetidae | *Peromyscus winkelmanni* | EN | Actual | 0.060 |
| Rodentia | Cricetidae | *Phyllotis definitus* | EN | Actual | 0.054 |
| Rodentia | Cricetidae | *Reithrodontomys bakeri* | EN | Actual | 0.071 |
| Rodentia | Cricetidae | *Sigmodon planifrons* | EN | Actual | 0.072 |
| Rodentia | Cricetidae | *Thomasomys hylophilus* | EN | Actual | 0.061 |
| Rodentia | Cricetidae | *Thomasomys monochromos* | EN | Actual | 0.044 |
| Rodentia | Cricetidae | *Wilfredomys oenax* | EN | Actual | 0.648 |
| Rodentia | Cricetidae | *Xenomys nelsoni* | EN | Actual | 0.509 |
| Rodentia | Ctenomyidae | *Ctenomys australis* | EN | Actual | 1.298 |
| Rodentia | Ctenomyidae | *Ctenomys bonettoi* | EN | Actual | 2.059 |
| Rodentia | Ctenomyidae | *Ctenomys flamarioni* | EN | Actual | 1.175 |
| Rodentia | Ctenomyidae | *Ctenomys occultus* | EN | Actual | 2.254 |
| Rodentia | Ctenomyidae | *Ctenomys pilarensis* | EN | Actual | 2.030 |
| Rodentia | Ctenomyidae | *Ctenomys rionegrensis* | EN | Actual | 1.047 |
| Rodentia | Diatomyidae | *Laonastes aenigmamus* | EN | Actual | 0.502 |
| Rodentia | Dipodidae | *Sicista kazbegica* | EN | Actual | 0.048 |
| Rodentia | Echimyidae | *Callistomys pictus* | EN | Actual | 0.400 |
| Rodentia | Echimyidae | *Phyllomys brasiliensis* | EN | Actual | 0.513 |
| Rodentia | Echimyidae | *Phyllomys lundi* | EN | Actual | 0.270 |
| Rodentia | Echimyidae | *Phyllomys thomasi* | EN | Actual | 0.743 |
| Rodentia | Echimyidae | *Trinomys eliasi* | EN | Actual | 0.882 |
| Rodentia | Echimyidae | *Trinomys moojeni* | EN | Actual | 0.245 |
| Rodentia | Echimyidae | *Trinomys yonenagae* | EN | Actual | 1.248 |
| Rodentia | Heteromyidae | *Dipodomys ingens* | EN | Actual | 0.425 |
| Rodentia | Heteromyidae | *Dipodomys stephensi* | EN | Actual | 0.378 |
| Rodentia | Heteromyidae | *Heteromys oasicus* | EN | Actual | 0.228 |
| Rodentia | Heteromyidae | *Liomys spectabilis* | EN | Actual | 0.119 |
| Rodentia | Muridae | *Apodemus gurkha* | EN | Actual | 0.047 |
| Rodentia | Muridae | *Batomys russatus* | EN | Actual | 0.320 |
| Rodentia | Muridae | *Bunomys prolatus* | EN | Actual | 0.060 |
| Rodentia | Muridae | *Chiropodomys karlkoopmani* | EN | Actual | 0.395 |
| Rodentia | Muridae | *Crateromys heaneyi* | EN | Actual | 0.223 |
| Rodentia | Muridae | *Crateromys schadenbergi* | EN | Actual | 0.074 |
| Rodentia | Muridae | *Dasymys montanus* | EN | Actual | 0.271 |
| Rodentia | Muridae | *Desmomys yaldeni* | EN | Actual | 0.234 |
| Rodentia | Muridae | *Echiothrix leucura* | EN | Actual | 0.133 |
| Rodentia | Muridae | *Grammomys gigas* | EN | Actual | 0.148 |
| Rodentia | Muridae | *Hapalomys longicaudatus* | EN | Actual | 0.860 |
| Rodentia | Muridae | *Hybomys badius* | EN | Actual | 0.247 |
| Rodentia | Muridae | *Hylomyscus baeri* | EN | Actual | 0.678 |
| Rodentia | Muridae | *Lamottemys okuensis* | EN | Actual | 0.511 |
| Rodentia | Muridae | *Leopoldamys siporanus* | EN | Actual | 0.395 |
| Rodentia | Muridae | *Lophuromys dieterleni* | EN | Actual | 0.057 |
| Rodentia | Muridae | *Lophuromys eisentrauti* | EN | Actual | 0.130 |
| Rodentia | Muridae | *Lophuromys rahmi* | EN | Actual | 0.163 |
| Rodentia | Muridae | *Mallomys gunung* | EN | Actual | 0.079 |
| Rodentia | Muridae | *Maxomys pagensis* | EN | Actual | 0.395 |
| Rodentia | Muridae | *Maxomys wattsi* | EN | Actual | 0.060 |
| Rodentia | Muridae | *Melomys aerosus* | EN | Actual | 0.060 |
| Rodentia | Muridae | *Melomys caurinus* | EN | Actual | 0.261 |
| Rodentia | Muridae | *Melomys matambuai* | EN | Actual | 1.039 |
| Rodentia | Muridae | *Melomys talaudium* | EN | Actual | 0.261 |
| Rodentia | Muridae | *Meriones dahli* | EN | Actual | 0.068 |
| Rodentia | Muridae | *Mus famulus* | EN | Actual | 0.285 |
| Rodentia | Muridae | *Nesokia bunnii* | EN | Actual | 2.803 |
| Rodentia | Muridae | *Notomys aquilo* | EN | Actual | 0.944 |
| Rodentia | Muridae | *Otomys burtoni* | EN | Actual | 0.060 |
| Rodentia | Muridae | *Paraleptomys rufilatus* | EN | Actual | 0.405 |
| Rodentia | Muridae | *Paramelomys gressitti* | EN | Actual | 0.055 |
| Rodentia | Muridae | *Paulamys naso* | EN | Actual | 0.069 |
| Rodentia | Muridae | *Pogonomys fergussoniensis* | EN | Actual | 0.242 |
| Rodentia | Muridae | *Praomys hartwigi* | EN | Actual | 0.239 |
| Rodentia | Muridae | *Praomys morio* | EN | Actual | 0.060 |
| Rodentia | Muridae | *Praomys obscurus* | EN | Actual | 0.224 |
| Rodentia | Muridae | *Pseudomys fumeus* | EN | Actual | 0.191 |
| Rodentia | Muridae | *Rattus hainaldi* | EN | Actual | 0.069 |
| Rodentia | Muridae | *Rattus lugens* | EN | Actual | 0.395 |
| Rodentia | Muridae | *Rattus simalurensis* | EN | Actual | 0.406 |
| Rodentia | Muridae | *Rattus vandeuseni* | EN | Actual | 0.090 |
| Rodentia | Muridae | *Solomys salebrosus* | EN | Actual | 0.459 |
| Rodentia | Muridae | *Solomys sapientis* | EN | Actual | 0.237 |
| Rodentia | Muridae | *Sundamys maxi* | EN | Actual | 0.105 |
| Rodentia | Muridae | *Tokudaia tokunoshimensis* | EN | Actual | 0.423 |
| Rodentia | Muridae | *Uromys neobritannicus* | EN | Actual | 0.218 |
| Rodentia | Muridae | *Uromys rex* | EN | Actual | 2.419 |
| Rodentia | Muridae | *Vandeleuria nilagirica* | EN | Actual | 0.263 |
| Rodentia | Nesomyidae | *Eliurus penicillatus* | EN | Actual | 0.229 |
| Rodentia | Nesomyidae | *Hypogeomys antimena* | EN | Actual | 0.896 |
| Rodentia | Nesomyidae | *Macrotarsomys ingens* | EN | Actual | 0.898 |
| Rodentia | Nesomyidae | *Mystromys albicaudatus* | EN | Actual | 0.568 |
| Rodentia | Nesomyidae | *Nesomys lambertoni* | EN | Actual | 0.443 |
| Rodentia | Sciuridae | *Ammospermophilus nelsoni* | EN | Actual | 0.550 |
| Rodentia | Sciuridae | *Cynomys parvidens* | EN | Actual | 0.314 |
| Rodentia | Sciuridae | *Hylopetes sipora* | EN | Actual | 0.432 |
| Rodentia | Sciuridae | *Iomys sipora* | EN | Actual | 0.432 |
| Rodentia | Sciuridae | *Marmota sibirica* | EN | Actual | 0.589 |
| Rodentia | Sciuridae | *Paraxerus vincenti* | EN | Actual | 0.291 |
| Rodentia | Sciuridae | *Petinomys lugens* | EN | Actual | 0.432 |
| Rodentia | Sciuridae | *Pteromyscus pulverulentus* | EN | Actual | 0.423 |
| Rodentia | Sciuridae | *Spermophilus atricapillus* | EN | Actual | 0.244 |
| Rodentia | Sciuridae | *Spermophilus brunneus* | EN | Actual | 0.325 |
| Rodentia | Sciuridae | *Spermophilus perotensis* | EN | Actual | 0.278 |
| Rodentia | Sciuridae | *Sundasciurus fraterculus* | EN | Actual | 0.395 |
| Rodentia | Spalacidae | *Spalax arenarius* | EN | Actual | 1.702 |
| Rodentia | Spalacidae | *Tachyoryctes macrocephalus* | EN | Actual | 0.150 |
| Scandentia | Tupaiidae | *Tupaia chrysogaster* | EN | Actual | 0.432 |
| Scandentia | Tupaiidae | *Tupaia nicobarica* | EN | Actual | 0.647 |
| Soricomorpha | Solenodontidae | *Solenodon cubanus* | EN | Actual | 0.109 |
| Soricomorpha | Solenodontidae | *Solenodon paradoxus* | EN | Actual | 0.300 |
| Soricomorpha | Soricidae | *Chimarrogale phaeura* | EN | Actual | 0.128 |
| Soricomorpha | Soricidae | *Crocidura ansellorum* | EN | Actual | 0.734 |
| Soricomorpha | Soricidae | *Crocidura baileyi* | EN | Actual | 0.173 |
| Soricomorpha | Soricidae | *Crocidura bottegoides* | EN | Actual | 0.289 |
| Soricomorpha | Soricidae | *Crocidura canariensis* | EN | Actual | 1.148 |
| Soricomorpha | Soricidae | *Crocidura desperata* | EN | Actual | 0.252 |
| Soricomorpha | Soricidae | *Crocidura hikmiya* | EN | Actual | 0.899 |
| Soricomorpha | Soricidae | *Crocidura lanosa* | EN | Actual | 0.181 |
| Soricomorpha | Soricidae | *Crocidura phaeura* | EN | Actual | 0.222 |
| Soricomorpha | Soricidae | *Crocidura picea* | EN | Actual | 0.213 |
| Soricomorpha | Soricidae | *Crocidura stenocephala* | EN | Actual | 0.156 |
| Soricomorpha | Soricidae | *Crocidura tansaniana* | EN | Actual | 0.200 |
| Soricomorpha | Soricidae | *Crocidura telfordi* | EN | Actual | 0.314 |
| Soricomorpha | Soricidae | *Crocidura thomensis* | EN | Actual | 0.909 |
| Soricomorpha | Soricidae | *Crocidura usambarae* | EN | Actual | 0.304 |
| Soricomorpha | Soricidae | *Cryptotis endersi* | EN | Actual | 0.077 |
| Soricomorpha | Soricidae | *Cryptotis mera* | EN | Actual | 0.278 |
| Soricomorpha | Soricidae | *Feroculus feroculus* | EN | Actual | 0.071 |
| Soricomorpha | Soricidae | *Myosorex blarina* | EN | Actual | 0.279 |
| Soricomorpha | Soricidae | *Myosorex kihaulei* | EN | Actual | 0.323 |
| Soricomorpha | Soricidae | *Myosorex okuensis* | EN | Actual | 0.201 |
| Soricomorpha | Soricidae | *Solisorex pearsoni* | EN | Actual | 0.200 |
| Soricomorpha | Soricidae | *Suncus fellowesgordoni* | EN | Actual | 0.314 |
| Soricomorpha | Soricidae | *Sylvisorex howelli* | EN | Actual | 0.259 |
| Soricomorpha | Soricidae | *Sylvisorex isabellae* | EN | Actual | 0.453 |
| Soricomorpha | Soricidae | *Sylvisorex morio* | EN | Actual | 0.060 |
| Artiodactyla | Bovidae | *Oryx dammah* | EW | Actual | 1.850 |
| Afrosoricida | Tenrecidae | *Hemicentetes nigriceps* | LC | Potential | 0.254 |
| Artiodactyla | Bovidae | *Capra pyrenaica* | LC | Potential | 0.291 |
| Artiodactyla | Bovidae | *Capra sibirica* | LC | Potential | 0.320 |
| Artiodactyla | Bovidae | *Capricornis crispus* | LC | Potential | 0.216 |
| Artiodactyla | Bovidae | *Capricornis swinhoei* | LC | Potential | 0.096 |
| Artiodactyla | Bovidae | *Cephalophus ogilbyi* | LC | Potential | 0.766 |
| Artiodactyla | Bovidae | *Gazella bennettii* | LC | Potential | 1.218 |
| Artiodactyla | Bovidae | *Ovibos moschatus* | LC | Potential | 1.267 |
| Artiodactyla | Bovidae | *Procapra gutturosa* | LC | Potential | 0.998 |
| Artiodactyla | Bovidae | *Pseudois nayaur* | LC | Potential | 0.329 |
| Artiodactyla | Bovidae | *Rupicapra pyrenaica* | LC | Potential | 0.073 |
| Artiodactyla | Camelidae | *Vicugna vicugna* | LC | Potential | 0.293 |
| Artiodactyla | Cervidae | *Cervus nippon* | LC | Potential | 0.285 |
| Artiodactyla | Cervidae | *Muntiacus atherodes* | LC | Potential | 0.668 |
| Artiodactyla | Cervidae | *Muntiacus muntjak* | LC | Potential | 0.685 |
| Artiodactyla | Cervidae | *Muntiacus reevesi* | LC | Potential | 0.523 |
| Artiodactyla | Tragulidae | *Tragulus napu* | LC | Potential | 0.690 |
| Carnivora | Canidae | *Vulpes rueppellii* | LC | Potential | 1.585 |
| Carnivora | Mephitidae | *Mydaus javanensis* | LC | Potential | 0.699 |
| Carnivora | Mephitidae | *Mydaus marchei* | LC | Potential | 0.331 |
| Carnivora | Mustelidae | *Mustela subpalmata* | LC | Potential | 1.318 |
| Carnivora | Procyonidae | *Procyon lotor* | LC | Potential | 1.135 |
| Carnivora | Viverridae | *Paradoxurus jerdoni* | LC | Potential | 0.328 |
| Carnivora | Viverridae | *Viverra tangalunga* | LC | Potential | 0.625 |
| Chiroptera | Emballonuridae | *Emballonura serii* | LC | Potential | 0.219 |
| Chiroptera | Emballonuridae | *Taphozous kapalgensis* | LC | Potential | 1.076 |
| Chiroptera | Hipposideridae | *Hipposideros sumbae* | LC | Potential | 0.159 |
| Chiroptera | Hipposideridae | *Hipposideros thomensis* | LC | Potential | 0.909 |
| Chiroptera | Mormoopidae | *Mormoops blainvillei* | LC | Potential | 0.645 |
| Chiroptera | Phyllostomidae | *Aethalops aequalis* | LC | Potential | 0.110 |
| Chiroptera | Phyllostomidae | *Ardops nichollsi* | LC | Potential | 0.123 |
| Chiroptera | Phyllostomidae | *Ariteus flavescens* | LC | Potential | 0.157 |
| Chiroptera | Phyllostomidae | *Brachyphylla cavernarum* | LC | Potential | 0.202 |
| Chiroptera | Phyllostomidae | *Carollia manu* | LC | Potential | 0.071 |
| Chiroptera | Phyllostomidae | *Monophyllus plethodon* | LC | Potential | 0.123 |
| Chiroptera | Pteropodidae | *Cynopterus nusatenggara* | LC | Potential | 0.181 |
| Chiroptera | Pteropodidae | *Dobsonia beauforti* | LC | Potential | 0.594 |
| Chiroptera | Pteropodidae | *Dobsonia crenulata* | LC | Potential | 0.268 |
| Chiroptera | Pteropodidae | *Dobsonia inermis* | LC | Potential | 0.660 |
| Chiroptera | Pteropodidae | *Dobsonia peronii* | LC | Potential | 0.181 |
| Chiroptera | Pteropodidae | *Dobsonia praedatrix* | LC | Potential | 0.253 |
| Chiroptera | Pteropodidae | *Melonycteris fardoulisi* | LC | Potential | 0.874 |
| Chiroptera | Pteropodidae | *Melonycteris woodfordi* | LC | Potential | 0.435 |
| Chiroptera | Pteropodidae | *Pteropus seychellensis* | LC | Potential | 0.142 |
| Chiroptera | Pteropodidae | *Pteropus tonganus* | LC | Potential | 0.195 |
| Chiroptera | Pteropodidae | *Rousettus celebensis* | LC | Potential | 0.270 |
| Chiroptera | Vespertilionidae | *Kerivoula kachinensis* | LC | Potential | 0.398 |
| Chiroptera | Vespertilionidae | *Pipistrellus wattsi* | LC | Potential | 3.617 |
| Dasyuromorphia | Dasyuridae | *Antechinus leo* | LC | Potential | 0.514 |
| Dasyuromorphia | Dasyuridae | *Myoictis wallacei* | LC | Potential | 1.353 |
| Dasyuromorphia | Dasyuridae | *Planigale novaeguineae* | LC | Potential | 1.304 |
| Dermoptera | Cynocephalidae | *Cynocephalus volans* | LC | Potential | 0.313 |
| Didelphimorphia | Didelphidae | *Thylamys cinderella* | LC | Potential | 0.504 |
| Diprotodontia | Macropodidae | *Dendrolagus lumholtzi* | LC | Potential | 0.328 |
| Diprotodontia | Macropodidae | *Dendrolagus spadix* | LC | Potential | 0.861 |
| Diprotodontia | Macropodidae | *Dorcopsis hageni* | LC | Potential | 0.671 |
| Diprotodontia | Macropodidae | *Dorcopsis muelleri* | LC | Potential | 0.768 |
| Diprotodontia | Macropodidae | *Dorcopsulus macleayi* | LC | Potential | 0.489 |
| Diprotodontia | Petauridae | *Petaurus biacensis* | LC | Potential | 0.442 |
| Diprotodontia | Phalangeridae | *Phalanger ornatus* | LC | Potential | 0.249 |
| Diprotodontia | Phalangeridae | *Phalanger rothschildi* | LC | Potential | 0.334 |
| Diprotodontia | Phalangeridae | *Strigocuscus pelengensis* | LC | Potential | 0.250 |
| Perissodactyla | Equidae | *Equus kiang* | LC | Potential | 0.430 |
| Primates | Atelidae | *Alouatta palliata* | LC | Potential | 0.555 |
| Primates | Atelidae | *Alouatta sara* | LC | Potential | 1.791 |
| Primates | Callitrichidae | *Callithrix geoffroyi* | LC | Potential | 0.367 |
| Primates | Callitrichidae | *Mico intermedius* | LC | Potential | 0.966 |
| Primates | Callitrichidae | *Mico manicorensis* | LC | Potential | 1.766 |
| Primates | Callitrichidae | *Mico mauesi* | LC | Potential | 1.210 |
| Primates | Callitrichidae | *Mico saterei* | LC | Potential | 1.381 |
| Primates | Callitrichidae | *Saguinus geoffroyi* | LC | Potential | 0.509 |
| Primates | Callitrichidae | *Saguinus martinsi* | LC | Potential | 1.170 |
| Primates | Cebidae | *Cebus capucinus* | LC | Potential | 0.506 |
| Primates | Cebidae | *Cebus cay* | LC | Potential | 1.302 |
| Primates | Cercopithecidae | *Cercopithecus campbelli* | LC | Potential | 16.352 |
| Primates | Cercopithecidae | *Chlorocebus aethiops* | LC | Potential | 0.962 |
| Primates | Cercopithecidae | *Colobus angolensis* | LC | Potential | 0.826 |
| Primates | Cercopithecidae | *Lophocebus albigena* | LC | Potential | 0.867 |
| Primates | Cercopithecidae | *Macaca cyclopis* | LC | Potential | 0.336 |
| Primates | Cercopithecidae | *Macaca fascicularis* | LC | Potential | 0.729 |
| Primates | Cercopithecidae | *Macaca fuscata* | LC | Potential | 0.228 |
| Primates | Cercopithecidae | *Miopithecus ogouensis* | LC | Potential | 0.671 |
| Primates | Cercopithecidae | *Presbytis rubicunda* | LC | Potential | 0.554 |
| Primates | Cercopithecidae | *Procolobus rufomitratus* | LC | Potential | 1.020 |
| Primates | Cercopithecidae | *Theropithecus gelada* | LC | Potential | 0.177 |
| Primates | Cheirogaleidae | *Cheirogaleus major* | LC | Potential | 0.255 |
| Primates | Cheirogaleidae | *Microcebus griseorufus* | LC | Potential | 1.017 |
| Primates | Cheirogaleidae | *Phaner furcifer* | LC | Potential | 0.257 |
| Primates | Cheirogaleidae | *Phaner pallescens* | LC | Potential | 0.769 |
| Primates | Galagonidae | *Euoticus pallidus* | LC | Potential | 0.749 |
| Primates | Galagonidae | *Galago matschiei* | LC | Potential | 0.393 |
| Primates | Indridae | *Avahi laniger* | LC | Potential | 0.248 |
| Primates | Loridae | *Arctocebus calabarensis* | LC | Potential | 0.728 |
| Primates | Pitheciidae | *Callicebus baptista* | LC | Potential | 1.766 |
| Primates | Pitheciidae | *Callicebus bernhardi* | LC | Potential | 1.520 |
| Primates | Pitheciidae | *Callicebus caligatus* | LC | Potential | 2.213 |
| Primates | Pitheciidae | *Callicebus pallescens* | LC | Potential | 2.179 |
| Rodentia | Capromyidae | *Capromys pilorides* | LC | Potential | 1.045 |
| Rodentia | Cricetidae | *Abrothrix markhami* | LC | Potential | 0.078 |
| Rodentia | Cricetidae | *Akodon torques* | LC | Potential | 0.081 |
| Rodentia | Cricetidae | *Andalgalomys olrogi* | LC | Potential | 1.213 |
| Rodentia | Cricetidae | *Dicrostonyx nelsoni* | LC | Potential | 1.007 |
| Rodentia | Cricetidae | *Euryoryzomys legatus* | LC | Potential | 0.561 |
| Rodentia | Cricetidae | *Euryoryzomys russatus* | LC | Potential | 0.423 |
| Rodentia | Cricetidae | *Handleyomys chapmani* | LC | Potential | 0.167 |
| Rodentia | Cricetidae | *Microtus abbreviatus* | LC | Potential | 0.955 |
| Rodentia | Cricetidae | *Microtus dogramacii* | LC | Potential | 0.506 |
| Rodentia | Cricetidae | *Microtus paradoxus* | LC | Potential | 0.309 |
| Rodentia | Cricetidae | *Oryzomys dimidiatus* | LC | Potential | 0.776 |
| Rodentia | Cricetidae | *Reithrodon typicus* | LC | Potential | 0.922 |
| Rodentia | Cricetidae | *Reithrodontomys rodriguezi* | LC | Potential | 0.070 |
| Rodentia | Cricetidae | *Tapecomys primus* | LC | Potential | 0.162 |
| Rodentia | Cricetidae | *Thomasomys eleusis* | LC | Potential | 0.067 |
| Rodentia | Cricetidae | *Thomasomys notatus* | LC | Potential | 0.084 |
| Rodentia | Cricetidae | *Thomasomys rhoadsi* | LC | Potential | 0.059 |
| Rodentia | Cricetidae | *Thomasomys taczanowskii* | LC | Potential | 0.085 |
| Rodentia | Cricetidae | *Thomasomys vestitus* | LC | Potential | 0.073 |
| Rodentia | Ctenomyidae | *Ctenomys goodfellowi* | LC | Potential | 1.032 |
| Rodentia | Ctenomyidae | *Ctenomys lewisi* | LC | Potential | 0.143 |
| Rodentia | Echimyidae | *Isothrix sinnamariensis* | LC | Potential | 0.680 |
| Rodentia | Echimyidae | *Proechimys semispinosus* | LC | Potential | 0.594 |
| Rodentia | Echimyidae | *Trinomys dimidiatus* | LC | Potential | 0.321 |
| Rodentia | Geomyidae | *Cratogeomys fumosus* | LC | Potential | 0.299 |
| Rodentia | Geomyidae | *Geomys texensis* | LC | Potential | 0.851 |
| Rodentia | Geomyidae | *Orthogeomys dariensis* | LC | Potential | 0.287 |
| Rodentia | Geomyidae | *Orthogeomys heterodus* | LC | Potential | 0.066 |
| Rodentia | Geomyidae | *Orthogeomys underwoodi* | LC | Potential | 0.291 |
| Rodentia | Geomyidae | *Thomomys bulbivorus* | LC | Potential | 0.827 |
| Rodentia | Geomyidae | *Thomomys townsendii* | LC | Potential | 0.605 |
| Rodentia | Heteromyidae | *Dipodomys merriami* | LC | Potential | 0.724 |
| Rodentia | Heteromyidae | *Dipodomys venustus* | LC | Potential | 47.726 |
| Rodentia | Heteromyidae | *Heteromys oresterus* | LC | Potential | 0.069 |
| Rodentia | Hystricidae | *Hystrix crassispinis* | LC | Potential | 0.668 |
| Rodentia | Hystricidae | *Hystrix javanica* | LC | Potential | 0.392 |
| Rodentia | Hystricidae | *Hystrix sumatrae* | LC | Potential | 0.804 |
| Rodentia | Muridae | *Apomys datae* | LC | Potential | 0.082 |
| Rodentia | Muridae | *Archboldomys musseri* | LC | Potential | 0.091 |
| Rodentia | Muridae | *Gerbillus juliani* | LC | Potential | 1.483 |
| Rodentia | Muridae | *Gerbillus rupicola* | LC | Potential | 2.352 |
| Rodentia | Muridae | *Lophuromys chrysopus* | LC | Potential | 0.209 |
| Rodentia | Muridae | *Lophuromys roseveari* | LC | Potential | 0.060 |
| Rodentia | Muridae | *Lophuromys woosnami* | LC | Potential | 0.344 |
| Rodentia | Muridae | *Maxomys alticola* | LC | Potential | 0.118 |
| Rodentia | Muridae | *Maxomys bartelsii* | LC | Potential | 0.131 |
| Rodentia | Muridae | *Melomys obiensis* | LC | Potential | 0.334 |
| Rodentia | Muridae | *Mus cypriacus* | LC | Potential | 0.362 |
| Rodentia | Muridae | *Mus mattheyi* | LC | Potential | 0.795 |
| Rodentia | Muridae | *Niviventer brahma* | LC | Potential | 0.148 |
| Rodentia | Muridae | *Niviventer coninga* | LC | Potential | 0.365 |
| Rodentia | Muridae | *Niviventer lepturus* | LC | Potential | 0.130 |
| Rodentia | Muridae | *Pseudomys johnsoni* | LC | Potential | 1.454 |
| Rodentia | Muridae | *Rattus baluensis* | LC | Potential | 0.054 |
| Rodentia | Muridae | *Rattus morotaiensis* | LC | Potential | 0.249 |
| Rodentia | Muridae | *Tarsomys apoensis* | LC | Potential | 0.173 |
| Rodentia | Nesomyidae | *Dendromus kivu* | LC | Potential | 0.194 |
| Rodentia | Nesomyidae | *Eliurus grandidieri* | LC | Potential | 0.243 |
| Rodentia | Sciuridae | *Eoglaucomys fimbriatus* | LC | Potential | 0.116 |
| Rodentia | Sciuridae | *Funisciurus carruthersi* | LC | Potential | 0.297 |
| Rodentia | Sciuridae | *Heliosciurus ruwenzorii* | LC | Potential | 0.327 |
| Rodentia | Sciuridae | *Marmota olympus* | LC | Potential | 0.151 |
| Rodentia | Sciuridae | *Prosciurillus rosenbergii* | LC | Potential | 0.219 |
| Rodentia | Sciuridae | *Sundasciurus hoogstraali* | LC | Potential | 0.433 |
| Rodentia | Sciuridae | *Sundasciurus steerii* | LC | Potential | 0.292 |
| Rodentia | Sciuridae | *Tamias alpinus* | LC | Potential | 0.066 |
| Rodentia | Sciuridae | *Tamias canipes* | LC | Potential | 0.539 |
| Rodentia | Sciuridae | *Tamias obscurus* | LC | Potential | 0.213 |
| Rodentia | Sciuridae | *Tamias ochrogenys* | LC | Potential | 0.220 |
| Scandentia | Tupaiidae | *Urogale everetti* | LC | Potential | 0.307 |
| Soricomorpha | Soricidae | *Crocidura arispa* | LC | Potential | 0.091 |
| Soricomorpha | Soricidae | *Crocidura cinderella* | LC | Potential | 1.788 |
| Soricomorpha | Soricidae | *Crocidura elgonius* | LC | Potential | 0.334 |
| Soricomorpha | Soricidae | *Crocidura nana* | LC | Potential | 0.446 |
| Soricomorpha | Soricidae | *Crocidura pachyura* | LC | Potential | 0.361 |
| Soricomorpha | Soricidae | *Crocidura ramona* | LC | Potential | 0.274 |
| Soricomorpha | Soricidae | *Cryptotis colombiana* | LC | Potential | 0.073 |
| Soricomorpha | Soricidae | *Cryptotis montivaga* | LC | Potential | 0.067 |
| Soricomorpha | Soricidae | *Cryptotis tamensis* | LC | Potential | 0.050 |
| Soricomorpha | Soricidae | *Sorex jacksoni* | LC | Potential | 2.067 |
| Soricomorpha | Soricidae | *Suncus madagascariensis* | LC | Potential | 0.495 |
| Soricomorpha | Soricidae | *Sylvisorex granti* | LC | Potential | 0.194 |
| Afrosoricida | Chrysochloridae | *Amblysomus septentrionalis* | NT | Potential | 0.433 |
| Afrosoricida | Tenrecidae | *Micropotamogale ruwenzorii* | NT | Potential | 0.213 |
| Artiodactyla | Bovidae | *Antilope cervicapra* | NT | Potential | 1.244 |
| Artiodactyla | Bovidae | *Capra cylindricornis* | NT | Potential | 0.086 |
| Artiodactyla | Bovidae | *Capricornis rubidus* | NT | Potential | 0.261 |
| Artiodactyla | Bovidae | *Hemitragus jemlahicus* | NT | Potential | 0.054 |
| Artiodactyla | Bovidae | *Naemorhedus goral* | NT | Potential | 0.154 |
| Artiodactyla | Bovidae | *Ovis ammon* | NT | Potential | 0.443 |
| Artiodactyla | Bovidae | *Procapra picticaudata* | NT | Potential | 0.390 |
| Artiodactyla | Cervidae | *Elaphodus cephalophus* | NT | Potential | 0.298 |
| Artiodactyla | Suidae | *Sus celebensis* | NT | Potential | 0.269 |
| Carnivora | Eupleridae | *Eupleres goudotii* | NT | Potential | 0.318 |
| Carnivora | Eupleridae | *Galidictis fasciata* | NT | Potential | 0.270 |
| Carnivora | Herpestidae | *Bdeogale jacksoni* | NT | Potential | 0.383 |
| Chiroptera | Hipposideridae | *Hipposideros doriae* | NT | Potential | 0.712 |
| Chiroptera | Pteropodidae | *Dobsonia pannietensis* | NT | Potential | 0.242 |
| Chiroptera | Pteropodidae | *Dyacopterus spadiceus* | NT | Potential | 0.615 |
| Chiroptera | Pteropodidae | *Pteropus chrysoproctus* | NT | Potential | 0.150 |
| Chiroptera | Pteropodidae | *Pteropus dasymallus* | NT | Potential | 0.307 |
| Chiroptera | Pteropodidae | *Pteropus pelewensis* | NT | Potential | 2940.530 |
| Chiroptera | Pteropodidae | *Pteropus rayneri* | NT | Potential | 0.700 |
| Chiroptera | Pteropodidae | *Pteropus samoensis* | NT | Potential | 0.167 |
| Chiroptera | Vespertilionidae | *Murina puta* | NT | Potential | 0.307 |
| Dasyuromorphia | Dasyuridae | *Antechinus godmani* | NT | Potential | 0.275 |
| Dasyuromorphia | Dasyuridae | *Dasyurus spartacus* | NT | Potential | 1.450 |
| Diprotodontia | Macropodidae | *Dendrolagus bennettianus* | NT | Potential | 0.247 |
| Diprotodontia | Macropodidae | *Macropus bernardus* | NT | Potential | 0.652 |
| Diprotodontia | Macropodidae | *Macropus parma* | NT | Potential | 0.214 |
| Diprotodontia | Macropodidae | *Petrogale burbidgei* | NT | Potential | 0.479 |
| Diprotodontia | Macropodidae | *Petrogale coenensis* | NT | Potential | 0.736 |
| Diprotodontia | Macropodidae | *Petrogale sharmani* | NT | Potential | 0.562 |
| Lagomorpha | Leporidae | *Lepus insularis* | NT | Potential | 0.051 |
| Pholidota | Manidae | *Manis culionensis* | NT | Potential | 0.331 |
| Primates | Callitrichidae | *Callithrix kuhlii* | NT | Potential | 0.296 |
| Primates | Callitrichidae | *Saguinus tripartitus* | NT | Potential | 1.382 |
| Primates | Cercopithecidae | *Macaca assamensis* | NT | Potential | 0.259 |
| Primates | Cercopithecidae | *Macaca thibetana* | NT | Potential | 0.300 |
| Primates | Cercopithecidae | *Papio papio* | NT | Potential | 1.973 |
| Primates | Cercopithecidae | *Presbytis femoralis* | NT | Potential | 0.735 |
| Primates | Cercopithecidae | *Presbytis siamensis* | NT | Potential | 0.756 |
| Primates | Cercopithecidae | *Procolobus verus* | NT | Potential | 0.802 |
| Primates | Cercopithecidae | *Trachypithecus cristatus* | NT | Potential | 0.727 |
| Primates | Cercopithecidae | *Trachypithecus obscurus* | NT | Potential | 0.551 |
| Primates | Cheirogaleidae | *Mirza coquereli* | NT | Potential | 0.723 |
| Primates | Daubentoniidae | *Daubentonia madagascariensis* | NT | Potential | 0.328 |
| Primates | Lemuridae | *Eulemur fulvus* | NT | Potential | 0.392 |
| Primates | Lemuridae | *Eulemur rufifrons* | NT | Potential | 0.763 |
| Primates | Lemuridae | *Lemur catta* | NT | Potential | 0.793 |
| Primates | Pitheciidae | *Callicebus nigrifrons* | NT | Potential | 0.389 |
| Primates | Tarsiidae | *Tarsius syrichta* | NT | Potential | 0.313 |
| Rodentia | Bathyergidae | *Cryptomys anselli* | NT | Potential | 0.976 |
| Rodentia | Capromyidae | *Mysateles prehensilis* | NT | Potential | 1.004 |
| Rodentia | Cricetidae | *Eothenomys wardi* | NT | Potential | 0.070 |
| Rodentia | Cricetidae | *Necromys obscurus* | NT | Potential | 0.977 |
| Rodentia | Cricetidae | *Peromyscus grandis* | NT | Potential | 0.099 |
| Rodentia | Cricetidae | *Phyllotis bonariensis* | NT | Potential | 0.479 |
| Rodentia | Cricetidae | *Rheomys thomasi* | NT | Potential | 0.228 |
| Rodentia | Ctenomyidae | *Ctenomys dorbignyi* | NT | Potential | 1.789 |
| Rodentia | Ctenomyidae | *Ctenomys emilianus* | NT | Potential | 0.120 |
| Rodentia | Dasyproctidae | *Dasyprocta guamara* | NT | Potential | 1.776 |
| Rodentia | Muridae | *Chrotomys gonzalesi* | NT | Potential | 0.065 |
| Rodentia | Muridae | *Grammomys dryas* | NT | Potential | 0.214 |
| Rodentia | Muridae | *Margaretamys elegans* | NT | Potential | 0.106 |
| Rodentia | Muridae | *Papagomys armandvillei* | NT | Potential | 0.126 |
| Rodentia | Muridae | *Rattus elaphinus* | NT | Potential | 0.250 |
| Rodentia | Muridae | *Rattus feliceus* | NT | Potential | 0.148 |
| Rodentia | Muridae | *Rattus jobiensis* | NT | Potential | 0.328 |
| Rodentia | Sciuridae | *Hylopetes nigripes* | NT | Potential | 0.331 |
| Rodentia | Sciuridae | *Lariscus obscurus* | NT | Potential | 0.395 |
| Rodentia | Sciuridae | *Paraxerus vexillarius* | NT | Potential | 0.299 |
| Rodentia | Sciuridae | *Sundasciurus moellendorffi* | NT | Potential | 0.421 |
| Soricomorpha | Soricidae | *Paracrocidura maxima* | NT | Potential | 0.184 |
| Afrosoricida | Chrysochloridae | *Carpitalpa arendsi* | VU | Actual | 0.168 |
| Afrosoricida | Chrysochloridae | *Chlorotalpa duthieae* | VU | Actual | 0.256 |
| Afrosoricida | Chrysochloridae | *Chrysospalax villosus* | VU | Actual | 0.266 |
| Afrosoricida | Chrysochloridae | *Neamblysomus julianae* | VU | Actual | 0.741 |
| Afrosoricida | Tenrecidae | *Microgale dryas* | VU | Actual | 0.227 |
| Afrosoricida | Tenrecidae | *Microgale monticola* | VU | Actual | 0.538 |
| Afrosoricida | Tenrecidae | *Microgale nasoloi* | VU | Actual | 0.553 |
| Artiodactyla | Bovidae | *Ammodorcas clarkei* | VU | Actual | 1.456 |
| Artiodactyla | Bovidae | *Ammotragus lervia* | VU | Actual | 1.073 |
| Artiodactyla | Bovidae | *Bison bonasus* | VU | Actual | 1.111 |
| Artiodactyla | Bovidae | *Bos gaurus* | VU | Actual | 0.489 |
| Artiodactyla | Bovidae | *Bos mutus* | VU | Actual | 0.494 |
| Artiodactyla | Bovidae | *Budorcas taxicolor* | VU | Actual | 0.122 |
| Artiodactyla | Bovidae | *Capra aegagrus* | VU | Actual | 0.389 |
| Artiodactyla | Bovidae | *Capra nubiana* | VU | Actual | 0.607 |
| Artiodactyla | Bovidae | *Capricornis sumatraensis* | VU | Actual | 0.245 |
| Artiodactyla | Bovidae | *Cephalophus zebra* | VU | Actual | 0.928 |
| Artiodactyla | Bovidae | *Dorcatragus megalotis* | VU | Actual | 0.342 |
| Artiodactyla | Bovidae | *Eudorcas rufifrons* | VU | Actual | 1.879 |
| Artiodactyla | Bovidae | *Gazella dorcas* | VU | Actual | 1.620 |
| Artiodactyla | Bovidae | *Gazella gazella* | VU | Actual | 0.919 |
| Artiodactyla | Bovidae | *Gazella subgutturosa* | VU | Actual | 1.259 |
| Artiodactyla | Bovidae | *Naemorhedus baileyi* | VU | Actual | 0.058 |
| Artiodactyla | Bovidae | *Naemorhedus caudatus* | VU | Actual | 0.238 |
| Artiodactyla | Bovidae | *Naemorhedus griseus* | VU | Actual | 0.199 |
| Artiodactyla | Bovidae | *Nanger soemmerringii* | VU | Actual | 0.789 |
| Artiodactyla | Bovidae | *Ovis orientalis* | VU | Actual | 0.755 |
| Artiodactyla | Bovidae | *Tetracerus quadricornis* | VU | Actual | 1.229 |
| Artiodactyla | Cervidae | *Blastocerus dichotomus* | VU | Actual | 1.038 |
| Artiodactyla | Cervidae | *Hippocamelus antisensis* | VU | Actual | 0.393 |
| Artiodactyla | Cervidae | *Hydropotes inermis* | VU | Actual | 1.114 |
| Artiodactyla | Cervidae | *Mazama bororo* | VU | Actual | 0.328 |
| Artiodactyla | Cervidae | *Mazama bricenii* | VU | Actual | 0.149 |
| Artiodactyla | Cervidae | *Mazama chunyi* | VU | Actual | 0.392 |
| Artiodactyla | Cervidae | *Mazama pandora* | VU | Actual | 1.313 |
| Artiodactyla | Cervidae | *Mazama rufina* | VU | Actual | 0.081 |
| Artiodactyla | Cervidae | *Muntiacus crinifrons* | VU | Actual | 0.300 |
| Artiodactyla | Cervidae | *Przewalskium albirostris* | VU | Actual | 0.209 |
| Artiodactyla | Cervidae | *Pudu mephistophiles* | VU | Actual | 0.064 |
| Artiodactyla | Cervidae | *Pudu puda* | VU | Actual | 0.176 |
| Artiodactyla | Cervidae | *Rucervus duvaucelii* | VU | Actual | 2.169 |
| Artiodactyla | Cervidae | *Rusa marianna* | VU | Actual | 0.238 |
| Artiodactyla | Cervidae | *Rusa timorensis* | VU | Actual | 0.144 |
| Artiodactyla | Cervidae | *Rusa unicolor* | VU | Actual | 0.741 |
| Artiodactyla | Hippopotamidae | *Hippopotamus amphibius* | VU | Actual | 1.122 |
| Artiodactyla | Moschidae | *Moschus moschiferus* | VU | Actual | 0.515 |
| Artiodactyla | Suidae | *Babyrousa babyrussa* | VU | Actual | 0.198 |
| Artiodactyla | Suidae | *Babyrousa celebensis* | VU | Actual | 0.222 |
| Artiodactyla | Suidae | *Sus ahoenobarbus* | VU | Actual | 0.336 |
| Artiodactyla | Suidae | *Sus barbatus* | VU | Actual | 0.507 |
| Artiodactyla | Suidae | *Sus philippensis* | VU | Actual | 0.239 |
| Carnivora | Ailuridae | *Ailurus fulgens* | VU | Actual | 0.084 |
| Carnivora | Eupleridae | *Cryptoprocta ferox* | VU | Actual | 0.531 |
| Carnivora | Eupleridae | *Mungotictis decemlineata* | VU | Actual | 1.262 |
| Carnivora | Eupleridae | *Salanoia concolor* | VU | Actual | 0.313 |
| Carnivora | Felidae | *Acinonyx jubatus* | VU | Actual | 1.657 |
| Carnivora | Felidae | *Felis nigripes* | VU | Actual | 1.619 |
| Carnivora | Felidae | *Leopardus guigna* | VU | Actual | 0.133 |
| Carnivora | Felidae | *Leopardus tigrinus* | VU | Actual | 1.059 |
| Carnivora | Felidae | *Neofelis diardi* | VU | Actual | 0.325 |
| Carnivora | Felidae | *Neofelis nebulosa* | VU | Actual | 0.241 |
| Carnivora | Felidae | *Panthera leo* | VU | Actual | 1.231 |
| Carnivora | Felidae | *Pardofelis marmorata* | VU | Actual | 0.310 |
| Carnivora | Felidae | *Prionailurus rubiginosus* | VU | Actual | 1.033 |
| Carnivora | Herpestidae | *Bdeogale omnivora* | VU | Actual | 0.827 |
| Carnivora | Herpestidae | *Herpestes fuscus* | VU | Actual | 0.555 |
| Carnivora | Herpestidae | *Liberiictis kuhni* | VU | Actual | 0.914 |
| Carnivora | Mephitidae | *Spilogale pygmaea* | VU | Actual | 0.497 |
| Carnivora | Mustelidae | *Aonyx cinerea* | VU | Actual | 0.669 |
| Carnivora | Mustelidae | *Lutrogale perspicillata* | VU | Actual | 0.972 |
| Carnivora | Mustelidae | *Martes gwatkinsii* | VU | Actual | 0.241 |
| Carnivora | Mustelidae | *Mustela felipei* | VU | Actual | 0.056 |
| Carnivora | Mustelidae | *Vormela peregusna* | VU | Actual | 1.010 |
| Carnivora | Ursidae | *Helarctos malayanus* | VU | Actual | 0.455 |
| Carnivora | Ursidae | *Melursus ursinus* | VU | Actual | 0.920 |
| Carnivora | Ursidae | *Tremarctos ornatus* | VU | Actual | 0.130 |
| Carnivora | Ursidae | *Ursus arctos* | VU | Actual | 0.805 |
| Carnivora | Ursidae | *Ursus maritimus* | VU | Actual | 1.194 |
| Carnivora | Ursidae | *Ursus thibetanus* | VU | Actual | 0.310 |
| Carnivora | Viverridae | *Arctictis binturong* | VU | Actual | 0.590 |
| Carnivora | Viverridae | *Chrotogale owstoni* | VU | Actual | 0.208 |
| Carnivora | Viverridae | *Diplogale hosei* | VU | Actual | 0.148 |
| Carnivora | Viverridae | *Genetta cristata* | VU | Actual | 1.013 |
| Carnivora | Viverridae | *Genetta johnstoni* | VU | Actual | 31.569 |
| Carnivora | Viverridae | *Hemigalus derbyanus* | VU | Actual | 0.694 |
| Carnivora | Viverridae | *Macrogalidia musschenbroekii* | VU | Actual | 0.229 |
| Carnivora | Viverridae | *Paradoxurus zeylonensis* | VU | Actual | 0.379 |
| Carnivora | Viverridae | *Viverra megaspila* | VU | Actual | 0.526 |
| Chiroptera | Craseonycteridae | *Craseonycteris thonglongyai* | VU | Actual | 1.067 |
| Chiroptera | Emballonuridae | *Balantiopteryx io* | VU | Actual | 0.623 |
| Chiroptera | Emballonuridae | *Taphozous hildegardeae* | VU | Actual | 0.509 |
| Chiroptera | Hipposideridae | *Coelops robinsoni* | VU | Actual | 0.401 |
| Chiroptera | Hipposideridae | *Hipposideros curtus* | VU | Actual | 0.587 |
| Chiroptera | Hipposideridae | *Hipposideros demissus* | VU | Actual | 0.211 |
| Chiroptera | Hipposideridae | *Hipposideros inornatus* | VU | Actual | 0.831 |
| Chiroptera | Hipposideridae | *Hipposideros khaokhouayensis* | VU | Actual | 0.277 |
| Chiroptera | Hipposideridae | *Hipposideros marisae* | VU | Actual | 0.909 |
| Chiroptera | Hipposideridae | *Hipposideros ridleyi* | VU | Actual | 0.517 |
| Chiroptera | Hipposideridae | *Hipposideros scutinares* | VU | Actual | 0.333 |
| Chiroptera | Hipposideridae | *Hipposideros sorenseni* | VU | Actual | 0.181 |
| Chiroptera | Hipposideridae | *Triaenops auritus* | VU | Actual | 0.258 |
| Chiroptera | Megadermatidae | *Macroderma gigas* | VU | Actual | 0.969 |
| Chiroptera | Molossidae | *Molossops aequatorianus* | VU | Actual | 1.413 |
| Chiroptera | Molossidae | *Mormopterus acetabulosus* | VU | Actual | 1.361 |
| Chiroptera | Molossidae | *Mormopterus minutus* | VU | Actual | 1.192 |
| Chiroptera | Molossidae | *Mormopterus norfolkensis* | VU | Actual | 0.334 |
| Chiroptera | Molossidae | *Mormopterus phrudus* | VU | Actual | 0.041 |
| Chiroptera | Molossidae | *Tadarida johorensis* | VU | Actual | 0.532 |
| Chiroptera | Molossidae | *Tadarida petersoni* | VU | Actual | 0.612 |
| Chiroptera | Molossidae | *Tomopeas ravus* | VU | Actual | 0.348 |
| Chiroptera | Mystacinidae | *Mystacina tuberculata* | VU | Actual | 0.152 |
| Chiroptera | Nycteridae | *Nycteris javanica* | VU | Actual | 0.439 |
| Chiroptera | Phyllostomidae | *Choeroniscus periosus* | VU | Actual | 0.505 |
| Chiroptera | Phyllostomidae | *Leptonycteris curasoae* | VU | Actual | 1.105 |
| Chiroptera | Phyllostomidae | *Leptonycteris yerbabuenae* | VU | Actual | 0.488 |
| Chiroptera | Phyllostomidae | *Lonchorhina orinocensis* | VU | Actual | 1.449 |
| Chiroptera | Phyllostomidae | *Musonycteris harrisoni* | VU | Actual | 0.242 |
| Chiroptera | Phyllostomidae | *Neonycteris pusilla* | VU | Actual | 1.640 |
| Chiroptera | Phyllostomidae | *Platyrrhinus ismaeli* | VU | Actual | 0.408 |
| Chiroptera | Phyllostomidae | *Stenoderma rufum* | VU | Actual | 0.207 |
| Chiroptera | Phyllostomidae | *Vampyressa melissa* | VU | Actual | 0.658 |
| Chiroptera | Pteropodidae | *Acerodon leucotis* | VU | Actual | 0.331 |
| Chiroptera | Pteropodidae | *Acerodon mackloti* | VU | Actual | 0.183 |
| Chiroptera | Pteropodidae | *Dobsonia emersa* | VU | Actual | 0.442 |
| Chiroptera | Pteropodidae | *Dyacopterus brooksi* | VU | Actual | 0.804 |
| Chiroptera | Pteropodidae | *Eidolon dupreanum* | VU | Actual | 0.520 |
| Chiroptera | Pteropodidae | *Harpyionycteris celebensis* | VU | Actual | 0.271 |
| Chiroptera | Pteropodidae | *Megaerops kusnotoi* | VU | Actual | 0.197 |
| Chiroptera | Pteropodidae | *Megaerops wetmorei* | VU | Actual | 0.266 |
| Chiroptera | Pteropodidae | *Myonycteris relicta* | VU | Actual | 0.536 |
| Chiroptera | Pteropodidae | *Notopteris macdonaldi* | VU | Actual | 0.167 |
| Chiroptera | Pteropodidae | *Notopteris neocaledonica* | VU | Actual | 0.197 |
| Chiroptera | Pteropodidae | *Nyctimene keasti* | VU | Actual | 0.188 |
| Chiroptera | Pteropodidae | *Nyctimene minutus* | VU | Actual | 0.150 |
| Chiroptera | Pteropodidae | *Pteropus anetianus* | VU | Actual | 0.132 |
| Chiroptera | Pteropodidae | *Pteropus lylei* | VU | Actual | 1.338 |
| Chiroptera | Pteropodidae | *Pteropus mahaganus* | VU | Actual | 0.481 |
| Chiroptera | Pteropodidae | *Pteropus molossinus* | VU | Actual | 0.415 |
| Chiroptera | Pteropodidae | *Pteropus ocularis* | VU | Actual | 0.150 |
| Chiroptera | Pteropodidae | *Pteropus ornatus* | VU | Actual | 0.215 |
| Chiroptera | Pteropodidae | *Pteropus poliocephalus* | VU | Actual | 0.365 |
| Chiroptera | Pteropodidae | *Pteropus rufus* | VU | Actual | 0.307 |
| Chiroptera | Pteropodidae | *Pteropus temminckii* | VU | Actual | 0.150 |
| Chiroptera | Pteropodidae | *Pteropus vetulus* | VU | Actual | 0.215 |
| Chiroptera | Pteropodidae | *Pteropus woodfordi* | VU | Actual | 0.999 |
| Chiroptera | Pteropodidae | *Rousettus bidens* | VU | Actual | 0.233 |
| Chiroptera | Pteropodidae | *Rousettus obliviosus* | VU | Actual | 0.142 |
| Chiroptera | Pteropodidae | *Rousettus spinalatus* | VU | Actual | 0.361 |
| Chiroptera | Pteropodidae | *Scotonycteris ophiodon* | VU | Actual | 0.676 |
| Chiroptera | Pteropodidae | *Styloctenium wallacei* | VU | Actual | 0.271 |
| Chiroptera | Pteropodidae | *Syconycteris carolinae* | VU | Actual | 0.249 |
| Chiroptera | Pteropodidae | *Syconycteris hobbit* | VU | Actual | 0.087 |
| Chiroptera | Rhinolophidae | *Rhinolophus canuti* | VU | Actual | 0.175 |
| Chiroptera | Rhinolophidae | *Rhinolophus guineensis* | VU | Actual | 0.564 |
| Chiroptera | Rhinolophidae | *Rhinolophus hillorum* | VU | Actual | 0.563 |
| Chiroptera | Rhinolophidae | *Rhinolophus mehelyi* | VU | Actual | 0.537 |
| Chiroptera | Rhinolophidae | *Rhinolophus ruwenzorii* | VU | Actual | 0.462 |
| Chiroptera | Vespertilionidae | *Arielulus societatis* | VU | Actual | 0.177 |
| Chiroptera | Vespertilionidae | *Chalinolobus tuberculatus* | VU | Actual | 0.215 |
| Chiroptera | Vespertilionidae | *Hesperoptenus tomesi* | VU | Actual | 0.539 |
| Chiroptera | Vespertilionidae | *Kerivoula flora* | VU | Actual | 0.195 |
| Chiroptera | Vespertilionidae | *Lasiurus degelidus* | VU | Actual | 0.157 |
| Chiroptera | Vespertilionidae | *Lasiurus insularis* | VU | Actual | 1.004 |
| Chiroptera | Vespertilionidae | *Lasiurus minor* | VU | Actual | 0.249 |
| Chiroptera | Vespertilionidae | *Murina aenea* | VU | Actual | 0.290 |
| Chiroptera | Vespertilionidae | *Murina rozendaali* | VU | Actual | 0.407 |
| Chiroptera | Vespertilionidae | *Myotis capaccinii* | VU | Actual | 0.399 |
| Chiroptera | Vespertilionidae | *Myotis dominicensis* | VU | Actual | 0.123 |
| Chiroptera | Vespertilionidae | *Myotis martiniquensis* | VU | Actual | 0.261 |
| Chiroptera | Vespertilionidae | *Myotis scotti* | VU | Actual | 0.290 |
| Chiroptera | Vespertilionidae | *Myotis sicarius* | VU | Actual | 0.748 |
| Chiroptera | Vespertilionidae | *Myotis vivesi* | VU | Actual | 1.461 |
| Chiroptera | Vespertilionidae | *Nyctalus furvus* | VU | Actual | 0.169 |
| Chiroptera | Vespertilionidae | *Plecotus sardus* | VU | Actual | 0.278 |
| Chiroptera | Vespertilionidae | *Rhogeessa minutilla* | VU | Actual | 1.048 |
| Chiroptera | Vespertilionidae | *Rhogeessa mira* | VU | Actual | 0.193 |
| Cinglulata | Dasypodidae | *Chaetophractus nationi* | VU | Actual | 0.525 |
| Cinglulata | Dasypodidae | *Dasypus pilosus* | VU | Actual | 0.075 |
| Cinglulata | Dasypodidae | *Priodontes maximus* | VU | Actual | 1.329 |
| Cinglulata | Dasypodidae | *Tolypeutes tricinctus* | VU | Actual | 0.697 |
| Dasyuromorphia | Dasyuridae | *Dasyuroides byrnei* | VU | Actual | 1.877 |
| Dasyuromorphia | Dasyuridae | *Murexia rothschildi* | VU | Actual | 0.117 |
| Dasyuromorphia | Dasyuridae | *Phascogale pirata* | VU | Actual | 0.997 |
| Dasyuromorphia | Dasyuridae | *Sminthopsis butleri* | VU | Actual | 0.822 |
| Dasyuromorphia | Dasyuridae | *Sminthopsis leucopus* | VU | Actual | 0.233 |
| Didelphimorphia | Didelphidae | *Chacodelphys formosa* | VU | Actual | 1.947 |
| Didelphimorphia | Didelphidae | *Marmosa xerophila* | VU | Actual | 0.851 |
| Didelphimorphia | Didelphidae | *Marmosops juninensis* | VU | Actual | 0.058 |
| Didelphimorphia | Didelphidae | *Micoureus phaeus* | VU | Actual | 0.153 |
| Didelphimorphia | Didelphidae | *Monodelphis umbristriata* | VU | Actual | 0.477 |
| Didelphimorphia | Didelphidae | *Thylamys karimii* | VU | Actual | 0.963 |
| Diprotodontia | Macropodidae | *Dendrolagus dorianus* | VU | Actual | 0.157 |
| Diprotodontia | Macropodidae | *Dendrolagus inustus* | VU | Actual | 0.515 |
| Diprotodontia | Macropodidae | *Dendrolagus stellarum* | VU | Actual | 0.173 |
| Diprotodontia | Macropodidae | *Dendrolagus ursinus* | VU | Actual | 0.161 |
| Diprotodontia | Macropodidae | *Dorcopsis luctuosa* | VU | Actual | 1.379 |
| Diprotodontia | Macropodidae | *Setonix brachyurus* | VU | Actual | 0.486 |
| Diprotodontia | Macropodidae | *Thylogale browni* | VU | Actual | 0.360 |
| Diprotodontia | Macropodidae | *Thylogale brunii* | VU | Actual | 1.231 |
| Diprotodontia | Phalangeridae | *Ailurops ursinus* | VU | Actual | 0.269 |
| Diprotodontia | Phalangeridae | *Spilocuscus papuensis* | VU | Actual | 0.395 |
| Diprotodontia | Phalangeridae | *Strigocuscus celebensis* | VU | Actual | 0.271 |
| Diprotodontia | Pseudocheiridae | *Pseudochirops coronatus* | VU | Actual | 0.084 |
| Diprotodontia | Pseudocheiridae | *Pseudochirulus schlegeli* | VU | Actual | 0.100 |
| Diprotodontia | Psuedocheiridae | *Pseudocheirus occidentalis* | VU | Actual | 0.566 |
| Erinaceomorpha | Erinaceidae | *Hylomys parvus* | VU | Actual | 0.103 |
| Lagomorpha | Leporidae | *Lepus castroviejoi* | VU | Actual | 0.102 |
| Lagomorpha | Leporidae | *Lepus corsicanus* | VU | Actual | 0.226 |
| Lagomorpha | Leporidae | *Lepus hainanus* | VU | Actual | 0.483 |
| Lagomorpha | Leporidae | *Nesolagus netscheri* | VU | Actual | 0.154 |
| Lagomorpha | Leporidae | *Sylvilagus transitionalis* | VU | Actual | 0.880 |
| Macroscelidea | Macroscelididae | *Rhynchocyon petersi* | VU | Actual | 0.506 |
| Paucituberculata | Caenolestidae | *Caenolestes convelatus* | VU | Actual | 0.067 |
| Peramelemorphia | Peramelidae | *Isoodon auratus* | VU | Actual | 0.562 |
| Peramelemorphia | Thylacomyidae | *Macrotis lagotis* | VU | Actual | 1.696 |
| Perissodactyla | Equidae | *Equus zebra* | VU | Actual | 0.786 |
| Perissodactyla | Rhinocerotidae | *Rhinoceros unicornis* | VU | Actual | 1.653 |
| Perissodactyla | Tapiridae | *Tapirus terrestris* | VU | Actual | 1.190 |
| Primates | Aotidae | *Aotus brumbacki* | VU | Actual | 1.504 |
| Primates | Aotidae | *Aotus griseimembra* | VU | Actual | 0.753 |
| Primates | Aotidae | *Aotus lemurinus* | VU | Actual | 0.097 |
| Primates | Aotidae | *Aotus miconax* | VU | Actual | 0.114 |
| Primates | Atelidae | *Alouatta belzebul* | VU | Actual | 0.983 |
| Primates | Atelidae | *Alouatta discolor* | VU | Actual | 0.914 |
| Primates | Atelidae | *Ateles paniscus* | VU | Actual | 0.998 |
| Primates | Atelidae | *Lagothrix lagotricha* | VU | Actual | 1.594 |
| Primates | Atelidae | *Lagothrix poeppigii* | VU | Actual | 1.676 |
| Primates | Callitrichidae | *Callibella humilis* | VU | Actual | 2.153 |
| Primates | Callitrichidae | *Callimico goeldii* | VU | Actual | 1.556 |
| Primates | Callitrichidae | *Callithrix aurita* | VU | Actual | 0.299 |
| Primates | Callitrichidae | *Mico leucippe* | VU | Actual | 0.782 |
| Primates | Callitrichidae | *Saguinus niger* | VU | Actual | 1.035 |
| Primates | Cebidae | *Saimiri oerstedii* | VU | Actual | 0.334 |
| Primates | Cebidae | *Saimiri vanzolinii* | VU | Actual | 1.933 |
| Primates | Cercopithecidae | *Cercocebus atys* | VU | Actual | 22.753 |
| Primates | Cercopithecidae | *Cercocebus torquatus* | VU | Actual | 0.868 |
| Primates | Cercopithecidae | *Cercopithecus erythrogaster* | VU | Actual | 1.172 |
| Primates | Cercopithecidae | *Cercopithecus erythrotis* | VU | Actual | 0.530 |
| Primates | Cercopithecidae | *Cercopithecus hamlyni* | VU | Actual | 0.896 |
| Primates | Cercopithecidae | *Cercopithecus lhoesti* | VU | Actual | 0.729 |
| Primates | Cercopithecidae | *Cercopithecus sclateri* | VU | Actual | 1.252 |
| Primates | Cercopithecidae | *Cercopithecus solatus* | VU | Actual | 0.389 |
| Primates | Cercopithecidae | *Chlorocebus djamdjamensis* | VU | Actual | 0.260 |
| Primates | Cercopithecidae | *Colobus polykomos* | VU | Actual | 29.178 |
| Primates | Cercopithecidae | *Colobus satanas* | VU | Actual | 0.756 |
| Primates | Cercopithecidae | *Colobus vellerosus* | VU | Actual | 0.723 |
| Primates | Cercopithecidae | *Macaca arctoides* | VU | Actual | 0.321 |
| Primates | Cercopithecidae | *Macaca hecki* | VU | Actual | 0.164 |
| Primates | Cercopithecidae | *Macaca leonina* | VU | Actual | 0.507 |
| Primates | Cercopithecidae | *Macaca nemestrina* | VU | Actual | 0.704 |
| Primates | Cercopithecidae | *Macaca nigrescens* | VU | Actual | 0.133 |
| Primates | Cercopithecidae | *Macaca ochreata* | VU | Actual | 0.274 |
| Primates | Cercopithecidae | *Macaca siberu* | VU | Actual | 0.395 |
| Primates | Cercopithecidae | *Macaca tonkeana* | VU | Actual | 0.246 |
| Primates | Cercopithecidae | *Mandrillus sphinx* | VU | Actual | 0.655 |
| Primates | Cercopithecidae | *Presbytis frontata* | VU | Actual | 0.396 |
| Primates | Cercopithecidae | *Presbytis hosei* | VU | Actual | 0.401 |
| Primates | Cercopithecidae | *Presbytis natunae* | VU | Actual | 1.226 |
| Primates | Cercopithecidae | *Presbytis thomasi* | VU | Actual | 0.316 |
| Primates | Cercopithecidae | *Semnopithecus hypoleucos* | VU | Actual | 0.388 |
| Primates | Cercopithecidae | *Trachypithecus auratus* | VU | Actual | 0.442 |
| Primates | Cercopithecidae | *Trachypithecus johnii* | VU | Actual | 0.258 |
| Primates | Cercopithecidae | *Trachypithecus laotum* | VU | Actual | 0.286 |
| Primates | Cercopithecidae | *Trachypithecus pileatus* | VU | Actual | 0.720 |
| Primates | Cheirogaleidae | *Phaner electromontis* | VU | Actual | 0.274 |
| Primates | Cheirogaleidae | *Phaner parienti* | VU | Actual | 0.161 |
| Primates | Hylobatidae | *Hoolock leuconedys* | VU | Actual | 0.334 |
| Primates | Indridae | *Propithecus deckenii* | VU | Actual | 0.657 |
| Primates | Indridae | *Propithecus verreauxi* | VU | Actual | 0.959 |
| Primates | Lemuridae | *Eulemur albifrons* | VU | Actual | 0.228 |
| Primates | Lemuridae | *Eulemur collaris* | VU | Actual | 0.229 |
| Primates | Lemuridae | *Eulemur coronatus* | VU | Actual | 0.310 |
| Primates | Lemuridae | *Eulemur mongoz* | VU | Actual | 0.814 |
| Primates | Lemuridae | *Eulemur rubriventer* | VU | Actual | 0.215 |
| Primates | Lemuridae | *Hapalemur griseus* | VU | Actual | 0.353 |
| Primates | Lemuridae | *Hapalemur meridionalis* | VU | Actual | 0.233 |
| Primates | Lemuridae | *Hapalemur occidentalis* | VU | Actual | 0.233 |
| Primates | Lepilemuridae | *Lepilemur edwardsi* | VU | Actual | 0.915 |
| Primates | Loridae | *Nycticebus bengalensis* | VU | Actual | 0.603 |
| Primates | Loridae | *Nycticebus coucang* | VU | Actual | 0.746 |
| Primates | Loridae | *Nycticebus menagensis* | VU | Actual | 0.672 |
| Primates | Loridae | *Nycticebus pygmaeus* | VU | Actual | 0.343 |
| Primates | Pitheciidae | *Cacajao ayresi* | VU | Actual | 2.247 |
| Primates | Pitheciidae | *Cacajao calvus* | VU | Actual | 1.809 |
| Primates | Pitheciidae | *Cacajao hosomi* | VU | Actual | 1.368 |
| Primates | Pitheciidae | *Callicebus medemi* | VU | Actual | 1.405 |
| Primates | Pitheciidae | *Callicebus melanochir* | VU | Actual | 0.418 |
| Primates | Pitheciidae | *Callicebus ornatus* | VU | Actual | 1.190 |
| Primates | Pitheciidae | *Callicebus personatus* | VU | Actual | 0.269 |
| Primates | Pitheciidae | *Pithecia albicans* | VU | Actual | 2.172 |
| Primates | Tarsiidae | *Tarsius bancanus* | VU | Actual | 0.707 |
| Primates | Tarsiidae | *Tarsius dentatus* | VU | Actual | 0.202 |
| Primates | Tarsiidae | *Tarsius tarsier* | VU | Actual | 0.291 |
| Rodentia | Bathyergidae | *Cryptomys kafuensis* | VU | Actual | 2.237 |
| Rodentia | Cricetidae | *Aepeomys reigi* | VU | Actual | 0.103 |
| Rodentia | Cricetidae | *Akodon latebricola* | VU | Actual | 0.059 |
| Rodentia | Cricetidae | *Akodon surdus* | VU | Actual | 0.059 |
| Rodentia | Cricetidae | *Alticola montosa* | VU | Actual | 0.145 |
| Rodentia | Cricetidae | *Anotomys leander* | VU | Actual | 0.065 |
| Rodentia | Cricetidae | *Arvicola sapidus* | VU | Actual | 0.501 |
| Rodentia | Cricetidae | *Calomys hummelincki* | VU | Actual | 1.468 |
| Rodentia | Cricetidae | *Dinaromys bogdanovi* | VU | Actual | 0.184 |
| Rodentia | Cricetidae | *Handleyomys rhabdops* | VU | Actual | 0.173 |
| Rodentia | Cricetidae | *Hylaeamys oniscus* | VU | Actual | 0.390 |
| Rodentia | Cricetidae | *Hyperacrius fertilis* | VU | Actual | 0.117 |
| Rodentia | Cricetidae | *Ichthyomys pittieri* | VU | Actual | 0.220 |
| Rodentia | Cricetidae | *Juliomys rimofrons* | VU | Actual | 0.881 |
| Rodentia | Cricetidae | *Mesocricetus auratus* | VU | Actual | 1.162 |
| Rodentia | Cricetidae | *Microakodontomys transitorius* | VU | Actual | 0.431 |
| Rodentia | Cricetidae | *Microtus breweri* | VU | Actual | 0.056 |
| Rodentia | Cricetidae | *Neotoma palatina* | VU | Actual | 0.111 |
| Rodentia | Cricetidae | *Nesoryzomys fernandinae* | VU | Actual | 0.167 |
| Rodentia | Cricetidae | *Nesoryzomys narboroughi* | VU | Actual | 0.246 |
| Rodentia | Cricetidae | *Neusticomys venezuelae* | VU | Actual | 0.523 |
| Rodentia | Cricetidae | *Pearsonomys annectens* | VU | Actual | 0.159 |
| Rodentia | Cricetidae | *Peromyscus simulus* | VU | Actual | 1.355 |
| Rodentia | Cricetidae | *Peromyscus zarhynchus* | VU | Actual | 0.233 |
| Rodentia | Cricetidae | *Phaenomys ferrugineus* | VU | Actual | 0.276 |
| Rodentia | Cricetidae | *Podomys floridanus* | VU | Actual | 1.654 |
| Rodentia | Cricetidae | *Proedromys bedfordi* | VU | Actual | 0.062 |
| Rodentia | Cricetidae | *Punomys kofordi* | VU | Actual | 0.098 |
| Rodentia | Cricetidae | *Punomys lemminus* | VU | Actual | 0.134 |
| Rodentia | Cricetidae | *Reithrodontomys hirsutus* | VU | Actual | 0.121 |
| Rodentia | Cricetidae | *Reithrodontomys microdon* | VU | Actual | 0.217 |
| Rodentia | Cricetidae | *Reithrodontomys tenuirostris* | VU | Actual | 0.148 |
| Rodentia | Cricetidae | *Sigmodon alleni* | VU | Actual | 0.307 |
| Rodentia | Cricetidae | *Sigmodon inopinatus* | VU | Actual | 0.065 |
| Rodentia | Cricetidae | *Thomasomys apeco* | VU | Actual | 0.050 |
| Rodentia | Cricetidae | *Thomasomys incanus* | VU | Actual | 0.057 |
| Rodentia | Cricetidae | *Thomasomys ischyrus* | VU | Actual | 0.074 |
| Rodentia | Cricetidae | *Thomasomys kalinowskii* | VU | Actual | 0.099 |
| Rodentia | Cricetidae | *Thomasomys macrotis* | VU | Actual | 0.050 |
| Rodentia | Cricetidae | *Thomasomys onkiro* | VU | Actual | 0.117 |
| Rodentia | Cricetidae | *Thomasomys pyrrhonotus* | VU | Actual | 0.078 |
| Rodentia | Ctenomyidae | *Ctenomys azarae* | VU | Actual | 0.829 |
| Rodentia | Ctenomyidae | *Ctenomys bergi* | VU | Actual | 0.363 |
| Rodentia | Ctenomyidae | *Ctenomys lami* | VU | Actual | 0.778 |
| Rodentia | Ctenomyidae | *Ctenomys latro* | VU | Actual | 0.657 |
| Rodentia | Ctenomyidae | *Ctenomys magellanicus* | VU | Actual | 0.385 |
| Rodentia | Ctenomyidae | *Ctenomys pundti* | VU | Actual | 1.574 |
| Rodentia | Dinomyidae | *Dinomys branickii* | VU | Actual | 1.404 |
| Rodentia | Dipodidae | *Allactaga tetradactyla* | VU | Actual | 1.399 |
| Rodentia | Dipodidae | *Sicista caucasica* | VU | Actual | 0.049 |
| Rodentia | Echimyidae | *Proechimys decumanus* | VU | Actual | 0.677 |
| Rodentia | Echimyidae | *Proechimys goeldii* | VU | Actual | 1.005 |
| Rodentia | Echimyidae | *Proechimys poliopus* | VU | Actual | 1.701 |
| Rodentia | Echimyidae | *Proechimys roberti* | VU | Actual | 1.006 |
| Rodentia | Erethizontidae | *Chaetomys subspinosus* | VU | Actual | 0.454 |
| Rodentia | Gliridae | *Myomimus roachi* | VU | Actual | 0.491 |
| Rodentia | Heteromyidae | *Chaetodipus dalquesti* | VU | Actual | 0.483 |
| Rodentia | Heteromyidae | *Chaetodipus goldmani* | VU | Actual | 0.859 |
| Rodentia | Heteromyidae | *Dipodomys elator* | VU | Actual | 1.886 |
| Rodentia | Heteromyidae | *Dipodomys nitratoides* | VU | Actual | 0.939 |
| Rodentia | Heteromyidae | *Heteromys teleus* | VU | Actual | 0.498 |
| Rodentia | Hystricidae | *Hystrix pumila* | VU | Actual | 0.331 |
| Rodentia | Muridae | *Archboldomys luzonensis* | VU | Actual | 0.305 |
| Rodentia | Muridae | *Bullimus gamay* | VU | Actual | 0.312 |
| Rodentia | Muridae | *Bunomys fratrorum* | VU | Actual | 0.146 |
| Rodentia | Muridae | *Crunomys melanius* | VU | Actual | 0.336 |
| Rodentia | Muridae | *Echiothrix centrosa* | VU | Actual | 0.151 |
| Rodentia | Muridae | *Eropeplus canus* | VU | Actual | 0.101 |
| Rodentia | Muridae | *Gerbillus hoogstraali* | VU | Actual | 0.516 |
| Rodentia | Muridae | *Grammomys minnae* | VU | Actual | 0.306 |
| Rodentia | Muridae | *Haeromys minahassae* | VU | Actual | 0.145 |
| Rodentia | Muridae | *Haeromys pusillus* | VU | Actual | 0.157 |
| Rodentia | Muridae | *Hapalomys delacouri* | VU | Actual | 0.250 |
| Rodentia | Muridae | *Hybomys lunaris* | VU | Actual | 0.279 |
| Rodentia | Muridae | *Komodomys rintjanus* | VU | Actual | 0.198 |
| Rodentia | Muridae | *Lophuromys medicaudatus* | VU | Actual | 0.216 |
| Rodentia | Muridae | *Lophuromys melanonyx* | VU | Actual | 0.204 |
| Rodentia | Muridae | *Margaretamys beccarii* | VU | Actual | 0.146 |
| Rodentia | Muridae | *Mastomys awashensis* | VU | Actual | 0.541 |
| Rodentia | Muridae | *Maxomys inflatus* | VU | Actual | 0.170 |
| Rodentia | Muridae | *Maxomys rajah* | VU | Actual | 0.704 |
| Rodentia | Muridae | *Maxomys whiteheadi* | VU | Actual | 0.704 |
| Rodentia | Muridae | *Mus mayori* | VU | Actual | 0.779 |
| Rodentia | Muridae | *Niviventer cameroni* | VU | Actual | 0.161 |
| Rodentia | Muridae | *Niviventer cremoriventer* | VU | Actual | 0.708 |
| Rodentia | Muridae | *Notomys cervinus* | VU | Actual | 1.920 |
| Rodentia | Muridae | *Notomys fuscus* | VU | Actual | 1.718 |
| Rodentia | Muridae | *Otomys lacustris* | VU | Actual | 0.466 |
| Rodentia | Muridae | *Otomys occidentalis* | VU | Actual | 0.230 |
| Rodentia | Muridae | *Phloeomys cumingi* | VU | Actual | 0.375 |
| Rodentia | Muridae | *Pithecheir melanurus* | VU | Actual | 0.126 |
| Rodentia | Muridae | *Praomys degraaffi* | VU | Actual | 0.226 |
| Rodentia | Muridae | *Pseudomys australis* | VU | Actual | 1.461 |
| Rodentia | Muridae | *Pseudomys calabyi* | VU | Actual | 0.956 |
| Rodentia | Muridae | *Pseudomys fieldi* | VU | Actual | 0.172 |
| Rodentia | Muridae | *Pseudomys novaehollandiae* | VU | Actual | 0.248 |
| Rodentia | Muridae | *Pseudomys oralis* | VU | Actual | 0.165 |
| Rodentia | Muridae | *Rattus hoogerwerfi* | VU | Actual | 0.091 |
| Rodentia | Muridae | *Rattus palmarum* | VU | Actual | 0.647 |
| Rodentia | Muridae | *Rattus richardsoni* | VU | Actual | 0.075 |
| Rodentia | Muridae | *Rattus satarae* | VU | Actual | 0.299 |
| Rodentia | Muridae | *Rattus stoicus* | VU | Actual | 0.457 |
| Rodentia | Muridae | *Rattus xanthurus* | VU | Actual | 0.146 |
| Rodentia | Muridae | *Rhynchomys isarogensis* | VU | Actual | 1.604 |
| Rodentia | Muridae | *Tarsomys echinatus* | VU | Actual | 0.132 |
| Rodentia | Muridae | *Thamnomys kempi* | VU | Actual | 0.156 |
| Rodentia | Muridae | *Thamnomys venustus* | VU | Actual | 0.375 |
| Rodentia | Muridae | *Uromys hadrourus* | VU | Actual | 0.256 |
| Rodentia | Muridae | *Xeromys myoides* | VU | Actual | 0.892 |
| Rodentia | Nesomyidae | *Delanymys brooksi* | VU | Actual | 0.172 |
| Rodentia | Nesomyidae | *Dendromus oreas* | VU | Actual | 0.060 |
| Rodentia | Nesomyidae | *Eliurus petteri* | VU | Actual | 0.211 |
| Rodentia | Octodontidae | *Octodon bridgesi* | VU | Actual | 0.232 |
| Rodentia | Octodontidae | *Octodon lunatus* | VU | Actual | 0.169 |
| Rodentia | Platacanthomyidae | *Platacanthomys lasiurus* | VU | Actual | 0.300 |
| Rodentia | Sciuridae | *Callosciurus adamsi* | VU | Actual | 0.585 |
| Rodentia | Sciuridae | *Callosciurus melanogaster* | VU | Actual | 0.395 |
| Rodentia | Sciuridae | *Funambulus layardi* | VU | Actual | 0.293 |
| Rodentia | Sciuridae | *Funambulus sublineatus* | VU | Actual | 0.275 |
| Rodentia | Sciuridae | *Hyosciurus ileile* | VU | Actual | 0.160 |
| Rodentia | Sciuridae | *Marmota menzbieri* | VU | Actual | 0.039 |
| Rodentia | Sciuridae | *Petaurista nobilis* | VU | Actual | 0.090 |
| Rodentia | Sciuridae | *Petinomys genibarbis* | VU | Actual | 0.630 |
| Rodentia | Sciuridae | *Petinomys setosus* | VU | Actual | 0.672 |
| Rodentia | Sciuridae | *Petinomys vordermanni* | VU | Actual | 0.372 |
| Rodentia | Sciuridae | *Rheithrosciurus macrotis* | VU | Actual | 0.668 |
| Rodentia | Sciuridae | *Rubrisciurus rubriventer* | VU | Actual | 0.271 |
| Rodentia | Sciuridae | *Spermophilus citellus* | VU | Actual | 0.892 |
| Rodentia | Sciuridae | *Spermophilus mohavensis* | VU | Actual | 0.437 |
| Rodentia | Sciuridae | *Spermophilus townsendii* | VU | Actual | 0.355 |
| Rodentia | Sciuridae | *Tamias bulleri* | VU | Actual | 0.147 |
| Rodentia | Spalacidae | *Spalax giganteus* | VU | Actual | 1.852 |
| Rodentia | Spalacidae | *Spalax zemni* | VU | Actual | 1.093 |
| Soricomorpha | Soricidae | *Crocidura allex* | VU | Actual | 0.249 |
| Soricomorpha | Soricidae | *Crocidura baluensis* | VU | Actual | 0.047 |
| Soricomorpha | Soricidae | *Crocidura eisentrauti* | VU | Actual | 0.137 |
| Soricomorpha | Soricidae | *Crocidura fumosa* | VU | Actual | 0.315 |
| Soricomorpha | Soricidae | *Crocidura glassi* | VU | Actual | 0.273 |
| Soricomorpha | Soricidae | *Crocidura macmillani* | VU | Actual | 0.143 |
| Soricomorpha | Soricidae | *Crocidura manengubae* | VU | Actual | 0.319 |
| Soricomorpha | Soricidae | *Crocidura zimmermanni* | VU | Actual | 0.132 |
| Soricomorpha | Soricidae | *Cryptotis gracilis* | VU | Actual | 0.069 |
| Soricomorpha | Soricidae | *Cryptotis griseoventris* | VU | Actual | 0.151 |
| Soricomorpha | Soricidae | *Cryptotis magna* | VU | Actual | 0.182 |
| Soricomorpha | Soricidae | *Cryptotis obscura* | VU | Actual | 0.160 |
| Soricomorpha | Soricidae | *Cryptotis phillipsii* | VU | Actual | 0.131 |
| Soricomorpha | Soricidae | *Myosorex longicaudatus* | VU | Actual | 0.226 |
| Soricomorpha | Soricidae | *Myosorex zinki* | VU | Actual | 0.124 |
| Soricomorpha | Soricidae | *Notiosorex villai* | VU | Actual | 0.220 |
| Soricomorpha | Soricidae | *Ruwenzorisorex suncoides* | VU | Actual | 0.219 |
| Soricomorpha | Soricidae | *Sorex macrodon* | VU | Actual | 0.311 |
| Soricomorpha | Soricidae | *Sorex milleri* | VU | Actual | 0.363 |
| Soricomorpha | Soricidae | *Suncus montanus* | VU | Actual | 0.253 |
| Soricomorpha | Soricidae | *Sylvisorex camerunensis* | VU | Actual | 0.216 |
| Soricomorpha | Soricidae | *Sylvisorex lunaris* | VU | Actual | 0.198 |
| Soricomorpha | Talpidae | *Desmana moschata* | VU | Actual | 1.165 |
| Soricomorpha | Talpidae | *Galemys pyrenaicus* | VU | Actual | 0.225 |
