# Supplementary material for: Geography of current and future global mammal extinction risk
Source: PLoS One. 2017 Nov 16;12(11):e0186934. doi: 10.1371/journal.pone.0186934 (PMC5690607; doi:10.1371/journal.pone.0186934)
Supplement: S2 Table — (DOCX) [file pone.0186934.s006.docx]

| **Accuracy Metric** | **Random Forest** |
| --- | --- |
| Out of bag (overall classification error rate) | 17.50% |
| Percentage of species correctly classified | 82.50% |
| Sensitivity (% of at risk species correctly classified) | 62.22% |
| Specificity (% of not at risk species correctly classified) | 88.20% |
| Cohen’s Kappa | 0.50 |
